# Syntheses and properties of thienyl-substituted dithienophenazines

**DOI:** 10.3762/bjoc.6.135

**Published:** 2010-12-13

**Authors:** Annemarie Meyer, Eva Sigmund, Friedhelm Luppertz, Gregor Schnakenburg, Immanuel Gadaczek, Thomas Bredow, Stefan-S Jester, Sigurd Höger

**Affiliations:** 1 Kekulé-Institut für Organische Chemie und Biochemie, Rheinische Friedrich-Wilhelms-Universität Bonn, Gerhard-Domagk-Str. 1, 53121 Bonn, Germany; 2 Institut für Anorganische Chemie, Rheinische Friedrich-Wilhelms-Universität Bonn, Gerhard-Domagk-Str. 1, 53121 Bonn, Germany; 3 Institut für Physikalische und Theoretische Chemie, Rheinische Friedrich-Wilhelms-Universität Bonn, Wegelerstr. 12, 53115 Bonn, Germany

**Keywords:** oligothiophenes, phenazines, scanning tunneling microscopy, self-assembled monolayers, TD-DFT calculations

## Abstract

A series of dithienophenazines with different lengths of the oligomeric thiophene units (quaterthiophenes and sexithiophenes) was synthesized. The thiophene and phenazine units act as electron donors and acceptors, respectively, resulting in characteristic absorption spectra. The optical spectra were calculated using time-dependent density functional theory at the B3LYP/TZVP level and verify the experimental data. Adsorption of the dithienophenazines on highly ordered pyrolytic graphite (HOPG) was investigated by scanning tunneling microscopy, showing that one of the compounds forms highly organized self-assembled monolayers.

## Introduction

Thiophene based oligomers and polymers have drawn considerable interest as active materials in various fields of organic electronics such as organic light-emitting diodes (OLEDs), organic thin-film transistors (OTFTs), or organic photovoltaics (OPVs) [1–3]. Concerning applications, organic materials are highly attractive due to their low cost and their particularly simple deposition from either vacuum or solution (casting or printing), and thus they are already utilized in industrial manufacturing. Particularly, deposition from the solution phase requires sufficiently high solubility, and one approach to cover this aspect is the functionalization with long, flexible alkyl substituents. Additionally, in many cases (short) oligomers are considerably better soluble than their corresponding (long) polymers, and can, as a matter of principle, be obtained in higher purity with respect to defects/polymerization faults. Moreover, they act as model systems allowing estimation of the pure polymer properties by extrapolation of the oligomer properties to infinite molecular weight [4].

Beside pure thiophene-based (well-established) materials, compounds with additional electron-poor moieties are in focus, as they are known to shift the HOMO and LUMO levels towards lower energies, thus increasing the compounds' stability against oxidation. In addition, the HOMO-LUMO gap is reduced (by the donor-acceptor (DA) approach), thereby red-shifting the absorption edge. This is of special importance, as there is still a need for new materials (exhibiting additionally the above mentioned processability criteria) absorbing the longer wavelength region (> 600 nm) of the sunlight spectrum, being required for photocurrent generation also from respective low-energy photons and thus enhancing the overall light harvesting yield in organic photovoltaics (OPV). For example, in the field of fused *N*- and *S*-heterocycles, thieno[3,4-*b*]pyrazines with various side groups have already found applications in OPVs [5–8]. In addition, fused bithiophenes with an enforced planarity, and therefore reduced optical gaps, and an increased π–π overlap of the polycyclic molecule backbone have entered various fields of organic electronics [9–13].

Apart from their optoelectronic properties, linear and branched as well as cyclic and polycyclic thiophenes are valuable templates for the epitaxial coadsorption of adlayers, in particular for fullerenes and metallacycles [14–17].

## Results and Discussion

Here we report the syntheses of two isomeric benzodithiophenediones, their respective phenazines and their coupling products with thiophene boronic acids. In both cases the thiophene units of the molecules act as electron rich parts (donors, D), whereas the phenazine moieties serve as electron deficient parts (acceptors, A), leading to a bathochromic shift of the UV–vis spectra in comparison with the non-condensed thiophene analogues. These assignments of the thiophene moieties as donors and the phenazine moieties as acceptors are confirmed by quantum-chemical calculations at the density-functional level.

Additionally, one of the compounds forms a self-assembled monolayer on a HOPG surface, as imaged by scanning tunneling microscopy (STM).

### Synthesis

**Scheme 1:**
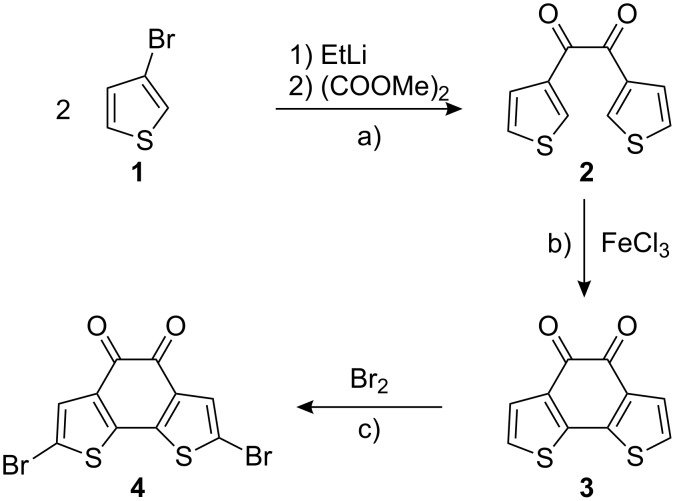
Synthesis of **4**. a) 1) EtLi, Et_2_O, −78 °C, 1 h; 2) (COOMe)_2_, Et_2_O, −78 °C, 2 h, 33%; b) FeCl_3_, MeNO_2_, CH_2_Cl_2_, rt, 16 h, 71%; c) Br_2_, AcOH, CHCl_3_, reflux, 5 h, 95%.

The synthesis of **4** is shown in Scheme 1. It is obtained in three steps from commercially available starting materials. The synthesis of **3** has previously been reported by benzoin condensation of the corresponding bithiophene derivative [18].

**Scheme 2:**
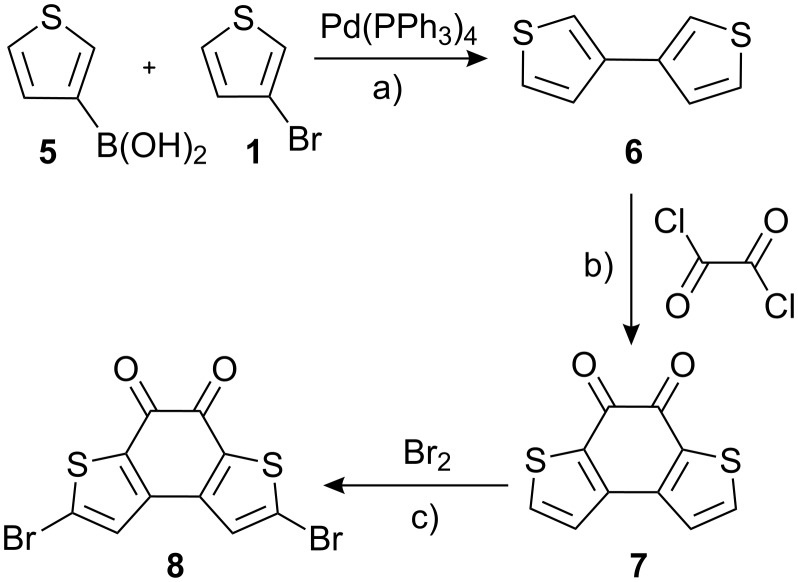
Synthesis of **8**. a) Pd(PPh_3_)_4_, Na_2_CO_3_, toluene, EtOH, H_2_O, reflux, 87%; b) oxalyl chloride, 1,2-dichloroethane, reflux, 85%; c) Br_2_, CHCl_3_, AcOH, reflux, 10 h, 97%.

Alternatively, we prepared first 3,3’-thenil (**2**) by metalation of 3-bromothiophene and reaction with dimethyl oxalate [19]. Subsequent oxidative intramolecular thiophene-thiophene coupling [20] with FeCl_3_ yielded **3** as a dark red (nearly black) solid. Bromination of **3** with bromine in acetic acid/chloroform gave **4** in nearly quantitative yield [21–22]. The respective regio isomer **8** was synthesized starting from commercially available 3-bromothiophene **1** and boronic acid **5** in three steps (Scheme 2). By Suzuki–Miyaura coupling we obtained 3,3’-bithiophene in good yields. The red diketone **7** was prepared by two-fold acylation with oxalyl chloride [23–24]. Bromination of **7** with NBS failed, however, the reaction with bromine under similar conditions as described for **4** afforded **8** in quantitative yields. Both routes towards the brominated bithiophene-diketones gave high overall yields and can be scaled up easily.

**Scheme 3:**
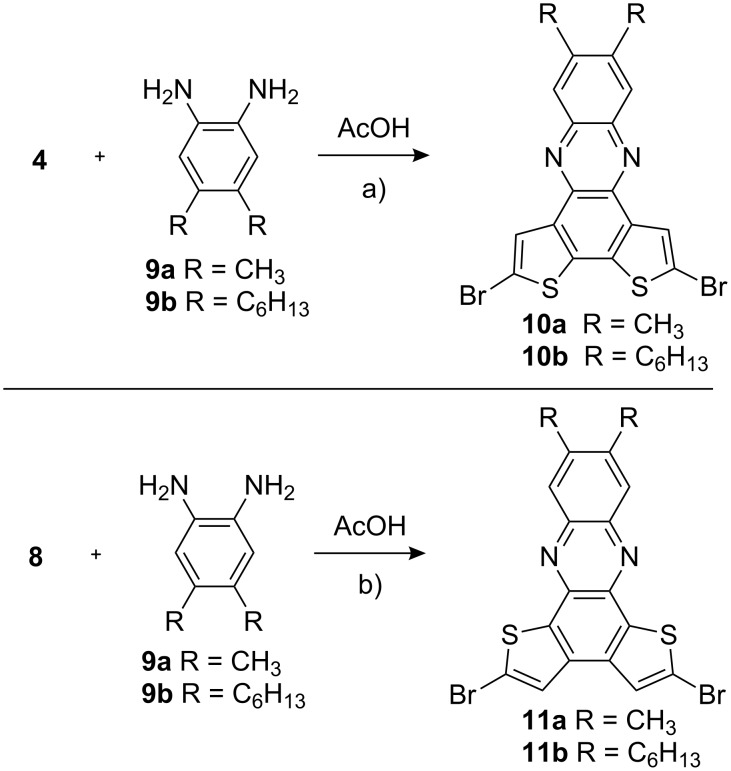
Preparation of phenazine isomers. a) AcOH, 50 °C, 2 h, 93% (**10a**), 53% (**10b**). b) AcOH, 50 °C, 2 h, 63% (**11a**), 50% (**11b**).

Condensation of the diketones **4** and **8** with diaminobenzenes **9a** and **9b**, containing methyl and hexyl side groups [25], in pure acetic acid at 50 °C gave the phenazines **10** and **11** as yellow solids in 50–93% yield (Scheme 3) [26]. The hexyl derivatives **10b** and **11b** showed a significantly higher solubility than the methyl analogues and could be more easily purified by column chromatography.

**Scheme 4:**
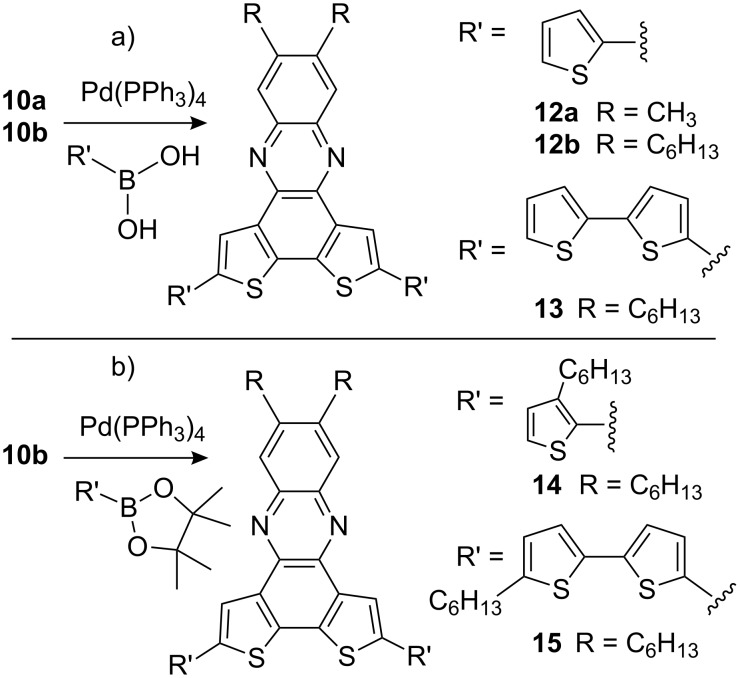
Suzuki–Miyaura reactions with **10a**/**10b**. a) Pd(PPh_3_)_4_, Na_2_CO_3_, toluene, EtOH, H_2_O, reflux, 23% (**12a**), 36% (**12b**), 26% (**13**); b) Pd(PPh_3_)_4_, 1 M Cs_2_CO_3_, toluene, reflux, 19% (**14**), Pd(PPh_3_)_4_, 2 M Na_2_CO_3_, toluene, Aliquat 336, reflux, 21% (**15**).

**Scheme 5:**
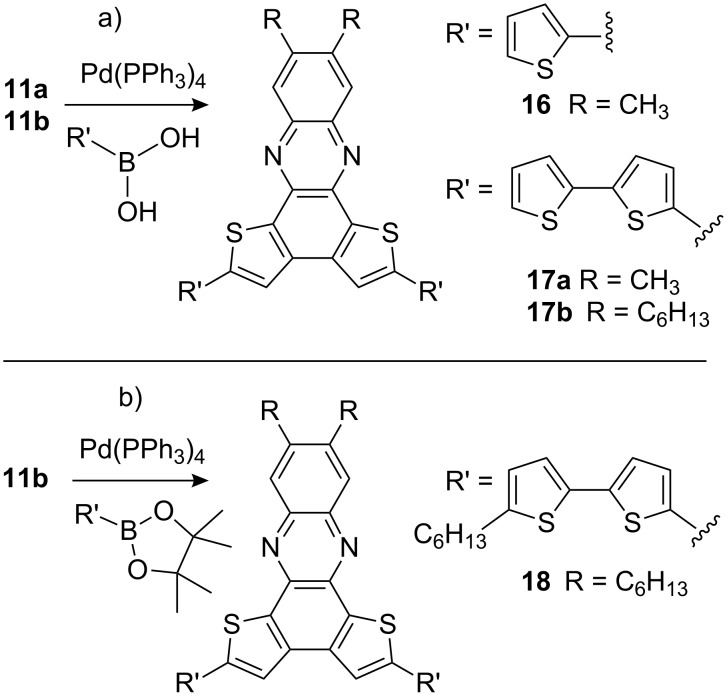
Suzuki–Miyaura reactions with **11a**/**11b**. a) Pd(PPh_3_)_4_, Na_2_CO_3_, toluene, EtOH, H_2_O, reflux, 50% (**16**), 15% (**17a**), 19% (**17b**); b) Pd(PPh_3_)_4_, 2 M Na_2_CO_3_, toluene, Aliquat 336, reflux, 56% (**18**).

Nevertheless, both compounds can serve as starting materials for thiophene oligomers with increased donor ability. Thiophene- and bithiophene boronic acids or esters were coupled via Suzuki–Miyaura reactions forming orange-colored thiophene-tetramers **12a**, **12b**, **14** and **16** and red-colored hexamers **13**, **15**, **17a**, **17b** and **18**, respectively (Scheme 4 and Scheme 5).

The low solubility of the methyl derivatives **12a**, **16** and **17a** made their purification tedious. However, this problem did not occur in the case of the hexyl substituted oligomers. Highly soluble compounds (20–50 mg/mL in chloroform or dichlorobenzene) were obtained by coupling of the hexyl substituted compounds **10b** and **11b** with alkyl substituted thiophene or bithiophene boronic acids (esters).

### UV–Vis spectra

**Figure 1:**
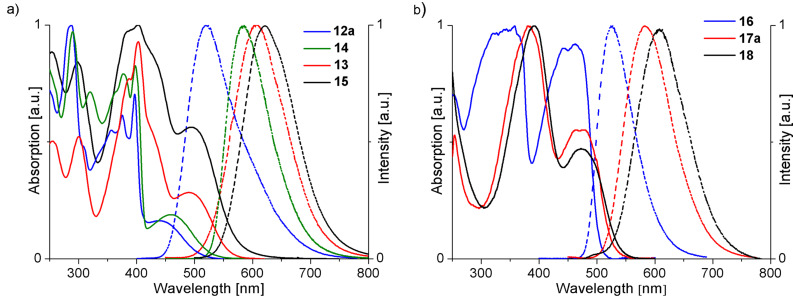
a) Absorption (solid lines) and emission (dotted lines) spectra of compounds **12a** (blue), **13** (red), **14** (green) and **15** (black) in solution (CH_2_Cl_2_). b) Absorption (solid lines) and emission (dotted lines) spectra of compounds **16** (blue), **17a** (red), **18** (black) in solution (CH_2_Cl_2_).

Figure 1a shows the UV–Vis absorption and emission spectra of **12a**, **13**, **14** and **15** in solution (CH_2_Cl_2_). In Figure 1b the spectra of the isomeric compounds **16**, **17a** and **18** are displayed [27]. All oligomers based on **10** exhibit strong absorption bands at ~250–350 nm and at ~350–450 nm with underlying vibronic structures. As expected, the longest wavelength absorption is for the hexathiophene derivatives shifted bathochromically in comparison to the tetrathiophene derivatives. The spectra of **16**, **17a** and **18** display two strong bands at ~300–400 nm and at ~400–500 nm, some of them show vibronic fine structures. The maxima of the emission spectra are shifted by ≈3500 to 4600 cm^−1^ with respect to the longest wavelength absorption maxima (Figure 1, Table 1).

**Table 1:** Spectral data of the synthesized oligomers. Absorption and emission spectra were measured in CH_2_Cl_2_ solutions. The zero–zero transition E_0–0_ values were estimated from the intersection of the absorption and emission spectra.

	λ_abs.max_ [nm]	λ_abs.onset_ [nm]	λ_abs.onset_ [eV]	λ_em.max_ [nm]	Stokes shift [cm^−1^]	E_0–0_ [nm]	E_0–0_ [eV]

**12a**	440	513	2.41	519	3459	463	2.68
**14**	462	550	2.25	583	4522	524	2.37
**13**	491	561	2.17	606	3865	531	2.33
**15**	495	577	2.13	622	4125	556	2.23

**16**	465	500	2.48	525	2457	493	2.51
**17a**	478	530	2.34	585	3833	520	2.38
**18**	476	539	2.30	610	4615	531	2.33

At the onset of the absorption, the zero–zero transition and the longest wavelength absorption maxima follow the same (expected) trends, that is the more extended π-systems (**13**, **15**, **17**, **18**) exhibit a smaller optical gap. Moreover, when compared with the optical spectra of the quater- and sexithiophene [28] (Table 2, **19** and **20**), a clear red-shift could be observed for both oligomer series, regardless if they are based on **10** or **11**, due to the intramolecular donor-acceptor character of the compounds.

**Table 2:** Spectral data of oligothiophenes in CH_2_Cl_2_._._

	λ_abs.max_ [nm]	λ_abs.onset_ [nm]	λ_abs.onset_ [eV]	λ_em.max_ [nm]	E_0–0_ [nm]	E_0–0_ [eV]

Quaterthiophene (**19**)	393	450	2.75	455, 478	440	2.82
Sexithiophene (**20**)	435	505	2.45	510, 537	488	2.54

### Quantum chemical calculations

To rationalize the observed trends in the optical absorption spectra of the oligomers, DFT calculations using the ORCA program package [29] were carried out. The ground state geometries of quaterthiophene **19**, the compounds **12a**, **16**, sexithiophene **20** and the compounds **13** and **17a** were optimized with the B3LYP functional [30] with a TZVP basis set [31]. Different rotations of the thiophene rings were investigated each corresponding to a different local minimum-energy structure. The differences in the total energy of the ground states are within the typical DFT error range of 3 to 11 kJ/mol. It can therefore be expected that at room temperature all conformations are present. For simplicity we only present results for the most stable structure. For the treatment of excited states time-dependent density functional theory (TD-DFT) was applied. The COSMO model was used to simulate the solution environment of the molecules in CH_2_Cl_2_ [32–33].

**Figure 2:**
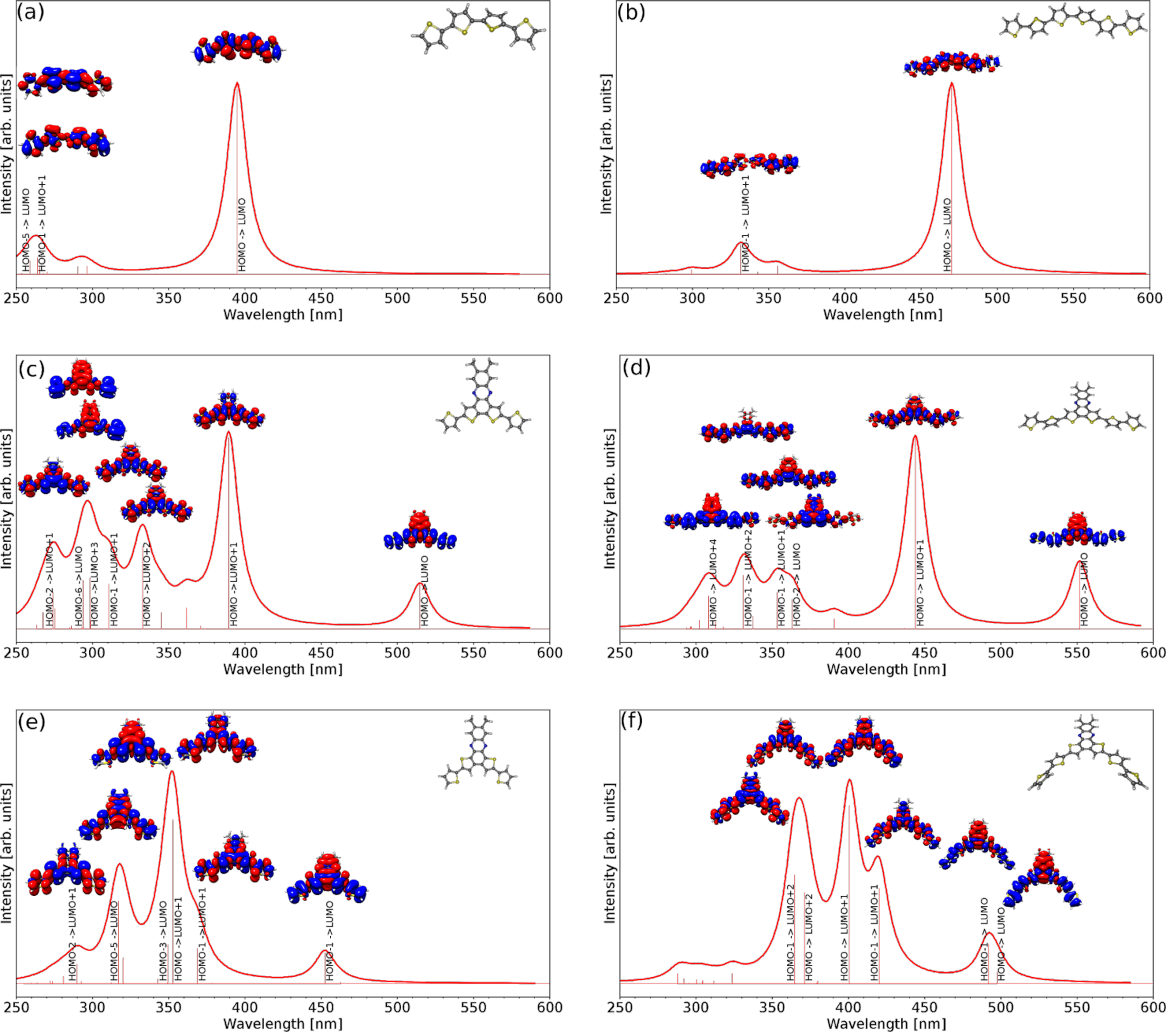
Calculated absorption spectra of (a) **19,** (b) **20**, (c) **12a**, (d) **13** with R=CH_3_, (e) **16** and (f) **17a**. In every spectrum the ground state geometry and the difference densities of the excitations are shown. Blue (red) color indicates a decrease (increase) of electron density after transition from the ground state to the excited state. Absorption signals with large oscillator strengths are labeled with the main excitation inducing the transition to this state.

The calculated spectra of the most stable conformers and the S_n_–S_0_ difference electron densities of selected compounds are shown in Figure 2. In general, the calculated excitation energies are in reasonable agreement with the experimental results (see also Table 3). It has to be kept in mind that the calculated values correspond to vertical excitation energies between electronic states while the measured optical spectra include vibronic effects.

**Table 3:** Lowest vertical excitation energies obtained by B3LYP/TZVP TD-DFT calculations of the quaterthiophene **19** and derivatives as well as sexithiophene **20** and derivatives (gas phase and in CH_2_Cl_2_) in eV, and oscillator strengths *f*_osc_.

State	**19**			**12a**			**16**		

	Gas	CH_2_Cl_2_	*f*_osc_	Gas	CH_2_Cl_2_	*f*_osc_	Gas	CH_2_Cl_2_	*f*_osc_

S_1_	3.14	3.13	1.39	2.37	2.11	0.19	2.69	2.56	0.26
S_2_	4.80	4.78	0.13	3.18	3.18	1.02	3.53	3.49	1.17

State	**20**			**13** (R=CH_3_)			**17a**		

	Gas	CH_2_Cl_2_	*f*_osc_	Gas	CH_2_Cl_2_	*f*_osc_	Gas	CH_2_Cl_2_	*f*_osc_

S_1_	2.64	2.63	2.27	2.21	1.97	0.45	2.43	2.21	0.08
S_2_	3.74	3.73	0.35	3.18	3.15	1.46	2.46	2.24	0.30

The dominating signals in the spectra of quaterthiophene **19** and sexithiophene **20** correspond to the HOMO-LUMO excitation. While the calculated S_1_ excitation energy of **19** differs by about 0.4 eV from the experimental result, the excitation energy of **20** is within the typical error range of B3LYP (Δ*E*_exp-calc_ = 0.1 eV) [34]. Both compounds have a similar spectrum, but the transitions of **20** are red shifted due to its larger delocalized π-system, which lowers the HOMO-LUMO gap as discussed above.

The difference density of **12a** (Figure 2c) shows that the S_0_-S_1_ excitation leads to an intramolecular charge transfer (CT) from the thiophenes to the phenazine part of the molecule. These calculations verify our initial assumption that the electron rich thiophene units act as electron donors and the electron poor phenazine unit as an electron acceptor in this intramolecular CT-complex. This CT state can also be observed in **16** (Figure 2e), but here the calculated difference between the S_1_ energies of **12a** and **16** (0.27 eV) is larger than the measured value (0.07 eV). Nevertheless, the computed excitation energies show a similar trend as the observed maxima of the absorption spectra.

The increased length of the thiophene chains in **13** compared to **12a** lowers the excitation energies of the CT states by about 0.2 eV due to an extended electron delocalization, as it is also experimentally observed. The same holds for the isomeric series. The calculated and observed optical gap in **17a** is considerably smaller than in **16**. It is worth noting that in both hexathiophene derivatives the charge reorganization upon excitation includes also the outermost thiophene rings, indicating that an extension of the chain would further reduce the optical gap. In addition, **17a** has a nearly degenerate S_2_ state, which also has CT properties. This HOMO-1 to LUMO excitation is visible in **17a** but not in **13** since the orbital energy and shape of HOMO and HOMO-1 in **17a** are nearly the same (Δ*E*_HOMO,HOMO-1_ =0.02 eV), while in **13** the energy difference between HOMO and HOMO-1 is larger than 0.6 eV [35].

### STM study

**Figure 3:**
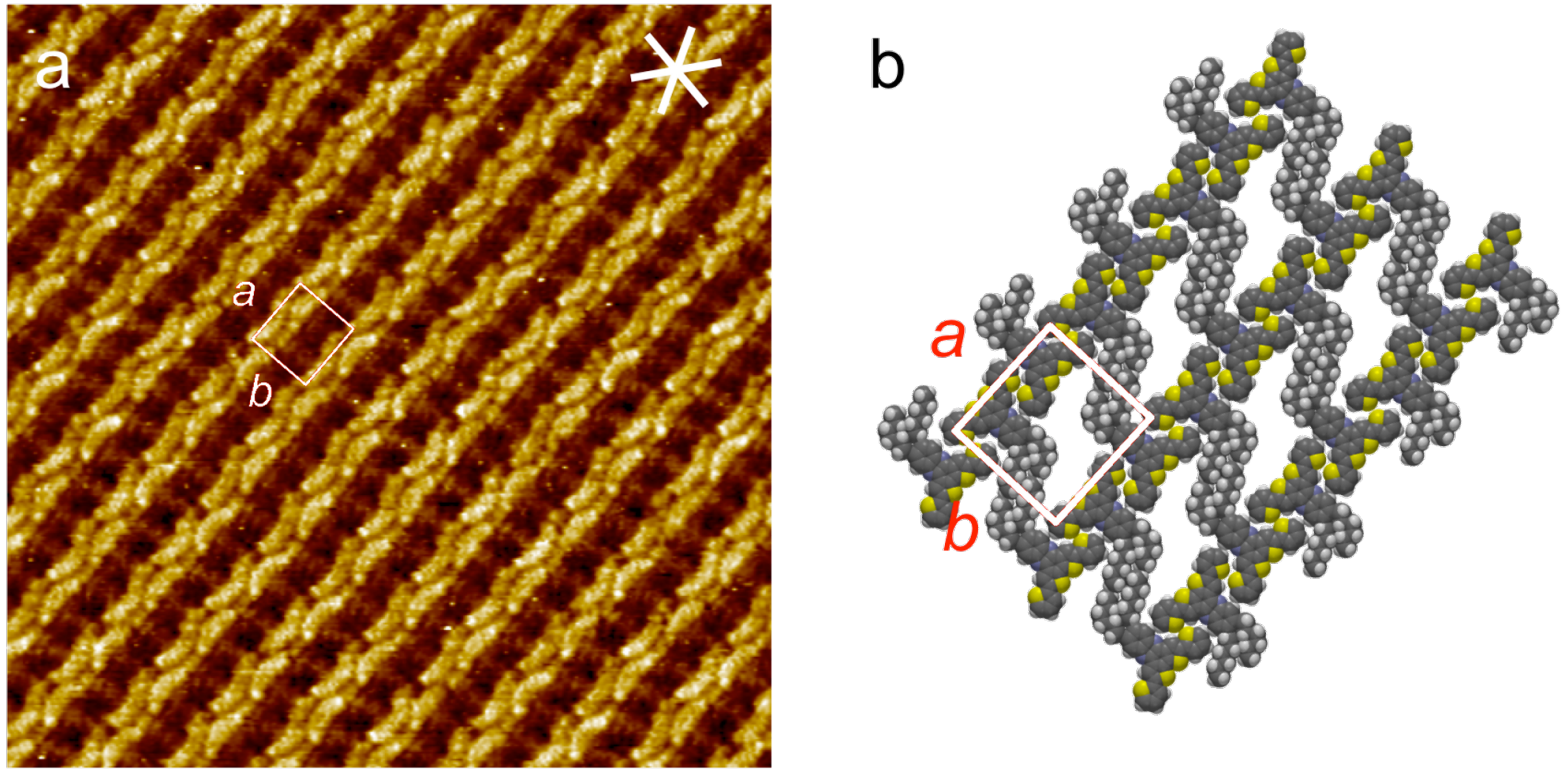
Self-assembled monolayer of **12b** on HOPG. a) STM image (*V*_S_ = −0.8 V, *I*_t_ = 80 pA, image size 25.0 × 25.0 nm^2^); b) structure model [39].

All synthesized thiophene oligomers **12**–**18** were investigated concerning their ability to form self-assembled monolayers (SAMs) at the HOPG/TCB interface (highly oriented pyrolytic graphite/1,2,4-trichlorobenzene). In situ STM was applied under typical conditions with typical tunneling parameters normally allowing the visualization of SAMs – if formed [36]. However, among all the substances only **12b** (10^−5^ M in TCB) assembles into crystalline monolayers on HOPG (Figure 3a). Bright and dark colors originate from locally high and low tunneling currents, resulting from unsaturated (backbone) and saturated (alkyl side chain) hydrocarbon segments, respectively [37]. A unit cell with *a* = 2.5 ± 0.1 nm, *b* = 2.3 ± 0.1 nm, *γ* = 90 ± 2° could be determined [38]. A proposed molecular model of the adsorbate geometry is shown in Figure 3b.

The banana-shaped bright features in Figure 3a are attributed to the backbones of **12b**, assembling in densely packed double rows. However, short alkyl chains are commonly not visible under the applied conditions. Nevertheless we assume they are oriented along the main crystallographic direction of the HOPG substrate.

Ordered adlayers were observed only for **12b**, but not for **12a**. Moreover, additional terminal alkyl chains terminating the end-capping thiophene substituents of **14**, **15** and **18** appear to hinder the formation of a self-assembled monolayer, most probably due to sterical hindrance of the molecules to cover the surface densely.

## Conclusion

We have developed a simple method for preparing phenazine-thiophene oligomers. These compounds show interesting optical characteristics, which can be tuned by attachment of different thiophene substituents. Currently we investigate the electrooptical properties of these compounds and similar derivatives. In addition, for one of the compounds a SAM on HOPG could be observed.

## Supporting Information

Experimental procedures and ^1^H and ^13^C NMR spectra and MS data for all new compounds are supplied, including X-ray data and a cif-file for **4** and **7**.

File 1 Syntheses and properties of thienyl-substituted dithienophenazines, experimental procedures, and characterization.

File 2 Syntheses and properties of thienyl-substituted dithienophenazines – cif files.
