# Supplementary material for: Syntheses and properties of thienyl-substituted dithienophenazines
Source: Beilstein J Org Chem. 2010 Dec 13;6:1180–7. doi: 10.3762/bjoc.6.135 (PMC3001992; doi:10.3762/bjoc.6.135)
Supplement: File 1 — Syntheses and properties of thienyl-substituted dithienophenazines, experimental procedures, and characterization. [file Beilstein_J_Org_Chem-06-1180-s001.pdf]

Supporting Information

for

Syntheses and properties of thienyl-substituted dithienophenazines

Annemarie Meyer[§1], Eva Sigmund[§1], Friedhelm Luppertz[1], Gregor Schnakenburg[2], Immanuel Gadaczek[3], Thomas Bredow*[3], Stefan-S. Jester[1] and Sigurd Höger *[1]

Address: [1]Kekulé-Institut für Organische Chemie und Biochemie, Rheinische Friedrich-Wilhelms-Universität Bonn, Gerhard-Domagk-Str. 1, 53121 Bonn, Germany; [2]Institut für Anorganische Chemie, Rheinische Friedrich-Wilhelms-Universität Bonn, Gerhard-Domagk-Str. 1, 53121 Bonn, Germany and [3]Institut für Physikalische und Theoretische Chemie, Rheinische Friedrich-Wilhelms-Universität Bonn, Wegelerstr. 12, 53115 Bonn, Germany

Email: Sigurd Höger - hoeger@uni-bonn.de; Thomas Bredow - bredow@thch.uni-bonn.de

*Corresponding author

[§]Both authors contributed equally to this publication

Table of contents

Experimental methods and characterization		S2
Figure 1	^{1}H NMR spectrum of **4**	S12
Figure 2	^{13}C NMR spectrum of **4**	S12
Figure 3	Crystal structures of **4**	S13
Crystal data of **4**		S14
Figure 4	Crystal structures of **7**	S15
Crystal data of **7**		S16
Figure 5	^{1}H NMR spectrum of **8**	S17
Figure 6	^{13}C NMR spectrum of **8**	S17
Figure 7	^{1}H NMR spectrum of **10a**	S18
Figure 8	^{1}H NMR spectrum of **10b**	S18
Figure 9	^{13}C NMR spectrum of **10b**	S19
Figure 10	^{1}H NMR spectrum of **11a**	S19
Figure 11	^{13}C NMR spectrum of **11a**	S20
Figure 12	^{1}H NMR spectrum of **11b**	S20
Figure 13	^{13}C NMR spectrum of **11b**	S21
Figure 14	^{1}H NMR spectrum of **12b**	S21
Figure 15	^{1}H NMR spectrum of **13**	S22
Figure 16	^{13}C NMR spectrum of **13**	S22
Figure 17	^{1}H NMR spectrum of **14**	S23
Figure 18	^{13}C NMR spectrum of **14**	S23
Figure 19	^{1}H NMR spectrum of **15**	S24
Figure 20	^{1}H NMR spectrum of **16**	S24
Figure 21	^{1}H NMR spectrum of **17a**	S25
Figure 22	^{1}H NMR spectrum of **17b**	S25
Figure 23	^{1}H NMR spectrum of **18**	S26
Figure 24	^{13}C NMR spectrum of **18**	S26
Figure 25	Absorption (solid line) and emission (dotted line) spectra of quaterthiophene (red) and sexithiophene (black) in CH_2Cl_2	S27
References		S27

Experimental section

Materials and equipment: All water and air sensitive manipulations were carried out under an argon atmosphere by standard Schlenk techniques. Tetrahydrofuran (THF) was distilled over Na and benzophenone under Ar. Dichloromethane (CH_2Cl_2) was distilled over CaH_2 under Ar. 3,3'-Thenil (**2**) [1], benzo[1,2-*b*:4,3-*b*']dithiophene-7,8-quinone (**7**) [2] and 1,2-diamino-4,5-dihexylbenzol (**9b**) [3] were prepared according to literature procedures. All other commercially available solvents and chemicals

were of reagent grade and used as received. ^{1}H NMR and ^{13}C NMR spectra were recorded on Bruker DPX 300, DPX 400 or DPX 500 spectrometers (300, 400 and 500 for ^{1}H and 75, 100 and 125 MHz for ^{13}C, respectively). Chemical shifts are given in parts per million (ppm) referring to tetramethylsilane (TMS). Mass spectra were measured on a Finnigan ThermoQuest MAT 95 XL (EI-MS), AEI MS-5 (EI-HRMS) and a Bruker Daltronics autoreflex TOF/TOF (MALDI-MS; matrix material: DCTB, no salts added). ICR MALDI data (recorded on a Bruker Daltonics Apex IV FT-ICR) are given for the high-molecular weight materials. *m/z* peaks smaller than 10% (compared to the basis peak) are not reported. UV–vis absorption spectra were recorded on a Shimadzu UV-2100 spectrometer with 10 mm quartz cuvettes. Fluorescence experiments were carried out on a Horiba Jobin Yvon FluoroMax-4 spectrofluorometer in all-transparent quartz cuvettes by monochromatic excitation at the indicated wavelength. All samples were prepared with appropriate concentrations in dichloromethane. Thin layer chromatography was conducted on silica gel coated aluminium plates (Macherey-Nagel, Alugramm SIL G/UV_{254} 0.25 mm coating with fluorescence indicator). Silica gel 60 M (Macherey-Nagel, 0.040–0.063 mm) was used as the stationary phase for column chromatography. A Shimadzu Recycling GPC System, equipped with a LC-20 AD pump, a SPD-20 A UV detector and a set of three preparative columns from PSS (10^{3} Å, 5 µ, 20 × 300 mm) was employed for purification.

STM investigations were performed, with an Agilent 5500 AFM/STM, operated in ambient conditions. Briefly, 2–4 µL of 10^{-4}–10^{-5} M solutions of the respective substance in 1,2,4-trichlorobenzene were dropped onto a freshly cleaved HOPG interface. All measurements were done in situ with bias voltages between −(0.4–1.6) V and tunneling currents in the range of 2–100 pA. In one case molecular self-assembly was observed.

Images were calibrated against a graphite atomic lattice using SPIP (ImageMetrology). Molecular geometries were calculated by force field methods, and adsorbate layers were simulated using Spartan 108 (Wavefunction, Inc.).

Crystal structure determination of 4 and 7: Suitable orange needle-like single crystals of **4** were grown from dichloromethane at ambient temperature. Suitable red plate-like single crystals of **7** were grown from dichloromethane at ambient temperature. The data collection of **4** was carried out on a STOE IPDS-2T diffractometer (area detector) equipped with a low-temperature device (Cryostream

700er series, Oxford Cryosystems, 123(2) K). The data collection of **7** was carried out on a Nonius KappaCCD diffractometer (area detector) equipped with a low-temperature device (Cryostream 600er series, Oxford Cryosystems, 123(2) K). Graphite monochromated Mo-K_α radiation (λ = 0.71073 Å) was used. Intensities were measured by fine-slicing ω- and φ-scans and corrected for background, polarization and Lorentz effects. Semi-empirical absorption corrections from equivalent reflections according to Blessing's method [4] were applied for the data sets. The structures were solved by direct methods and refined anisotropically by the least-squares procedure implemented in the SHELX program system [5]. The hydrogen atoms were included isotropically using the riding model on the bound carbon atoms. The illustrations of the molecular structures were prepared with Diamond 2.1c [6] CCDC 771987 (**4**), and CCDC 771988 (**7**) contain the supplementary crystallographic data for this paper. These data can be obtained free of charge from The Cambridge Crystallographic Data Centre via www.ccdc.cam.ac.uk/data_request/cif.

3,3'-Thenil (2): A solution of ethyllithium (59 mL, 1.7 M in dibutyl ether, 0.1 mol) in dry diethyl ether (200 mL) under argon was cooled to −78 °C and stirred. 3-Bromothiophene (**1**) (16.3 g, 0.1 mol) was added drop wise. Then, a solution of dimethyl oxalate (5.9 g, 0.05 mol) in 150 mL dry diethyl ether was added drop wise via a transfer cannula and the solution was stirred for 2 h at −78 °C. The reaction mixture was warmed to 0 °C and quenched by slowly adding an aqueous sulphuric acid solution (5%). The organic layer separated and was washed successfully with aqueous Na_2CO_3 solution (5%), water and brine, then dried over $MgSO_4$, filtered and the solvents were removed under vacuum. Purification of the crude product on silica gel (petroleum ether 40/60:CH_2Cl_2 = 1:1, R_f = 0.49) gave 3.71 g (0.033 mol, 33%) of **2** as a yellow solid. ^{1}H NMR (400 MHz, $CDCl_3$) δ ppm 8.36 (dd, J = 4.0 Hz, J = 1.6 Hz, 2H), 7.70 (dd, J = 6.8 Hz, J = 1.6 Hz, 2H), 7.39 (dd, J = 6.8 Hz, J = 1.6 Hz, 2H). ^{13}C NMR (75 MHz, $CDCl_3$) δ ppm 185.7, 137.6, 137.7, 127.5, 126.9. MS: (EI) *m/z* (%) = 222.0 (11) [$M^{+\cdot}$], 111.0 (100), 83 (10). EI HRMS: *m/z* (M^+) (calcd: 221.9809 g/mol) found: 221.9806. Chemical formula: $C_{10}H_6O_2S_2$. M_w: 222.28 g/mol.

Benzo[2,1-*b*:3,4-*b*']dithiophene-7,8-quinone (3): A solution of 3,3'-thenil (**2**) (570 mg, 2.6 mmol) in dry dichloromethane (150 mL) was stirred vigorously under argon at rt. A solution of $FeCl_3$ (880 mg, 5.4 mmol) in nitromethane (25 mL) was

added drop wise and the mixture was stirred over night. Water was added, the organic layer was separated and washed with saturated aqueous NH_4Cl solution and brine, dried over $MgSO_4$, filtered and the solvents were removed under vacuum. Purification of the crude product on silica gel with CH_2Cl_2 as eluent (R_f = 0.32) gave 405 mg (1.8 mmol, 71%) of **3** as a black solid. ^{1}H NMR (400 MHz, $CDCl_3$) δ ppm 7.51 (d, J = 4.4 Hz, 2H), 7.22 (d, J = 4.4 Hz, 2H). ^{13}C NMR (125 MHz, $CDCl_3$) δ ppm 174.6, 143.9, 135.1, 127.8, 125.6. MS (EI): m/z (%) = 219.9 (77) [$M^{+\cdot}$], 192.0 (100), 164.0 (15). EI HRMS: m/z (M^+) (calcd: 219.9652 g/mol) found: 219.9649. Chemical formula: $C_{10}H_4O_2S_2$. M_w: 220.27 g/mol.

2,5-Dibrombenzo[2,1-*b*:3,4-*b*']dithiophene-7,8-quinone (4): 3 (400 mg, 1.8 mmol) was dissolved in chloroform (9.5 mL) and acetic acid (10 mL) and cooled to 0 °C. A solution of bromine (595 mg, 3.7 mmol) diluted 1:10 (vol.) in chloroform was slowly added. After complete addition the reaction mixture was heated to reflux for 5 h. After cooling to rt the mixture was diluted with aqueous $Na_2S_2O_3$ solution. The organic layer was separated and washed successfully with saturated aqueous $NaHCO_3$ solution and brine, then dried over $MgSO_4$, filtered and concentrated under vacuum. Column chromatography of the crude product on silica gel with CH_2Cl_2 as eluent (R_f = 0.45) gave 650 mg (1.7 mmol, 95%) of **4** as a black solid. ^{1}H NMR (400 MHz, $CDCl_3$) δ ppm 7.46 (s, 2H). ^{13}C NMR (100 MHz, $CDCl_3$) δ ppm 172.6, 143.6, 135.4, 130.1, 114.6. MS (EI): m/z (%) = 375.8 (45) [$M^{+\cdot}$], 349.8 (100), 270.8 (62), 161.9 (53), 81.0 (16). EI HRMS: m/z (M^+) (calcd: 375.7863 g/mol) found: 375.7867. Chemical formula: $C_{10}H_2Br_2O_2S_2$. M_w: 378.06 g/mol.

3,3'-Bithiophene (6): 5 (3.96 g, 30.93 mmol), **1** (4.58 g, 28.10 mmol), Na_2CO_3 (17.82 g, 168.10 mmol), toluene (140 mL), ethanol (35 mL) and water (35 mL) were purged with argon for 1.5–2 h. $Pd(PPh_3)_4$ (320 mg, 0.28 mmol) was added and the reaction mixture was heated to reflux for 20 h, cooled to rt, poured into water and extracted with dichloromethane. The organic layer was washed successfully with water (2×), saturated aqueous $NaHCO_3$ solution and brine, then dried over $MgSO_4$, filtered and concentrated on a rotary evaporator. The crude product was purified by column chromatography on silica gel (petroleum ether 40/60:CH_2Cl_2 = 2:1, R_f = 0.60) to give 4.06 g (24.40 mmol, 87%) of **6** as colorless crystals. ^{1}H NMR (400 MHz, $CDCl_3$) δ ppm 7.38 (dd, J = 1.46 Hz, J = 2.79 Hz, 2H), 7.35 (dd, J = 2.90 Hz, J = 5.05

Hz, 2H), 7.34 (dd, J = 1.49 Hz, J = 5.03 Hz, 2H). ^{13}C NMR (100 MHz, $CDCl_3$) δ ppm 137.2, 126.3, 126.0, 119.7. MS (EI): *m/z* (%) = 165.9 (100) [$M^{+\cdot}$], 134.0 (10), 121.0 (25), 82.9 (10). EI HRMS: *m/z* (M^+) (calcd: 165.9905 g/mol) found: 162.9911. Chemical formula: $C_8H_6S_2$. M_w: 166.26 g/mol.

Benzo[1,2-*b*:4,3-*b'*]dithiophen-7,8-quinone (7): To a solution of **6** (1.96 g, 11.79 mmol) in 1,2-dichloroethane (25 mL) oxalyl chloride (0.60 mL, 6.99 mmol) was added. The reaction mixture was stirred for 5 d under reflux. Then another portion of oxalyl chloride (0.60 mL, 6.99 mmol) was added and the solution was stirred for additional 5 d under reflux. The mixture was cooled to rt and stored over night. A red precipitate was collected and washed with *n*-hexane and warm ethanol to afford 2.20 g (9.98 mmol, 85%) of **7**. ^{1}H NMR (400 MHz, $CDCl_3$) δ ppm 7.82 (d, J = 4.97 Hz, 2H), 7.29 (d, J = 4.97 Hz, 2H). ^{13}C NMR (100 MHz, $CDCl_3$) δ ppm 173.8, 142.4, 138.5, 135.0, 124.8. MS: (EI) *m/z* (%) = 219.9 (75) [$M^{+\cdot}$], 191.9 (100), 163.9 (25). EI HRMS: *m/z* (M^+) (calcd: 219.9647 g/mol) found: 219.9651. Chemical formula: $C_{10}H_4O_2S_2$. M_w: 220.27 g/mol.

2,5-Dibromo-benzo[1,2-*b*:4,3-*b'*]dithiophene-7,8-quinone (8): The synthesis was performed analogous to the synthesis of **4** (CH_2Cl_2, R_f = 0.56). **8** was obtained as a red solid (yield: 97%). ^{1}H NMR (400 MHz, $CDCl_3$) δ ppm 7.23 (s, 2H). ^{13}C NMR (100 MHz, $CDCl_3$) δ ppm 173.9, 141.5, 138.3, 128.8, 127.8. MS (EI): *m/z* (%) = 375.8 (40) [$M^{+\cdot}$], 349.8 (100), 321.8 (10), 242.8 (15), 161.9 (20), 80.9 (15). EI HRMS: *m/z* (M^+) (calcd: 375.7857 g/mol) found: 375.7864. Chemical formula: $C_{10}H_2Br_2O_2S_2$. M_w: 380.08 g/mol.

2,5-Dibromo-9,10-dimethyldithieno[3,2-*a*:2',3'-*c*]phenazine (10a): A solution of **4** (267 mg, 0.7 mmol) and **9a** (88 mg, 0.6 mmol) in acetic acid (25 mL) was stirred at 50 °C for 2 h and over night at rt. The yellow precipitate was filtered and washed with methanol. The crude product was purified by chromatography on silica gel (petroleum ether 40/60:CH_2Cl_2 = 2:1, R_f = 0.68) to yield 310 mg (0.7 mmol, 93%) of **10a** as a yellow solid. ^{1}H NMR (400 MHz, $CDCl_3$) δ ppm 8.49 (s, 2H), 8.12 (s, 2H), 2.61 (s, 6H). MS (EI): *m/z* (%) = 475.9 (47) [$M^{+\cdot}$], 462.9 (11), 396.9 (11). EI HRMS: *m/z* (M^+) (calcd: 475.8647 g/mol) found: 475.8651. Chemical formula: $C_{18}H_{10}Br_2N_2S_2$. M_w: 478.22 g/mol.

2,5-Dibrom-9,10-dihexyldithieno[3,2-*a*:2',3'-*c*]phenazine (10b): The synthesis was carried out analogous to the synthesis of **10a** (petroleum ether 40/60:CH_2Cl_2 = 1:1, R_f = 0.76). **10b** was obtained as an orange solid (yield: 53%). ^{1}H NMR (400 MHz, $CDCl_3$) δ ppm 8.36 (s, 2H), 8.03 (s, 2H), 2.89 (t, J = 7.8 Hz, 4H), 1.84–1.76 (m, 4H), 1.56–1.48 (m, 4H), 1.41–1.35 (m, 8H), 0.94 (t, J = 7.2 Hz, 6H). ^{13}C NMR (75 MHz, $CDCl_3$) δ ppm 145.3, 140.7, 137.4, 134. 8, 134.6, 127.4, 127.1, 112.9, 33.0, 31.8, 30.3, 29.5, 22.7, 14.1. MS (EI): *m/z* (%) = 616.0 (49) [$M^{+\cdot}$], 476.8 (31). ICR MALDI: *m/z* (M^+) (calcd: 616.0212 g/mol) found: 616.0289. Chemical formula: $C_{28}H_{30}Br_2N_2S_2$. M_w: 618.49 g/mol.

2,5-Dibromo-9,10-dimethyldithieno[2,3-*a*:3',2'-*c*]phenazine (11a): The synthesis was carried out analogous to the synthesis of **10a** (petroleum ether 40/60:CH_2Cl_2 = 2:1, R_f = 0.52). **11a** was obtained as a yellow solid (yield: 50%). ^{1}H NMR (400 MHz, $CDCl_3$) δ ppm 8.01 (s, 2H), 7.66 (s, 2H), 2.57 (s, 6H). ^{13}C NMR (100 MHz, $CDCl_3$) δ ppm 167.8, 161.2, 155.2, 140.9, 136.4, 128.2, 126.0, 125.4, 20.6. MS (EI): *m/z* (%) = 475.9 (100) [$M^{+\cdot}$], 460.9 (10), 394.8 (5). EI HRMS: *m/z* (M^+) (calcd: 475.8647 g/mol) found: 475.8652. Chemical formula: $C_{18}H_{10}Br_2N_2S_2$. M_w: 478.22 g/mol.

2,5-Dibromo-9,10-dihexyldithieno[2,3-*a*:3',2'-*c*]phenazine (11b): The synthesis was carried out analogous to the synthesis of **10a** (petroleum ether 40/60:CH_2Cl_2 = 2:1, R_f = 0.25). **11b** was obtained as a yellow solid (yield: 63%). ^{1}H NMR (400 MHz, $CDCl_3$) δ ppm 7.98 (s, 2H), 7.59 (s, 2H), 2.87 (t, J = 7.7 Hz, 4H), 1.83–1.74 (m, 4H), 1.55–1.46 (m, 4H), 1.45–1.33 (m, 8H), 0.93 (t, J = 7.1 Hz, 6H). ^{13}C NMR (75 MHz, $CDCl_3$) δ ppm 144.9, 141.0, 139.2, 136.1, 135.6, 129.8, 127.2, 123.1, 33.0, 31.8, 30.4, 29.46, 22.6, 14.1. MS (EI): *m/z* (%) = 616.0 (50) [$M^{+\cdot}$], 538.1 (20), 402.0 (40). EI HRMS: *m/z* (M^+) (calcd: 616.0212 g/mol) found: 616.0211. Chemical formula: $C_{28}H_{30}Br_2N_2S_2$. M_w: 616.02 g/mol.

2,5-Dithienyl-9,10-dimethyldithieno[3,2-*a*:2',3'-*c*]phenazine (12a): The synthesis was carried out analogous to the synthesis of **6** (petroleum ether 40/60:CH_2Cl_2 = 1:1, R_f = 0.28). **12a** was obtained as an orange solid (yield: 23%). NMR measurements were not possible due to its low solubility. MS (EI): *m/z* (%) = 484.0 (100) [$M^{+\cdot}$], 402.0 (7), 242.0 (14). EI HRMS: *m/z* (M^+) (calcd: 484.0196 g/mol) found: 484.0203. Chemical formula: $C_{26}H_{16}N_2S_4$. M_w: 484.68 g/mol.

2,5-Dithienyl-9,10-dihexyldithieno[3,2-*a*:2',3'-*c*]phenazine (12b): The synthesis was carried out analogous to the synthesis of **6**. Purification of the crude product on silica gel (petroleum ether 40/60:CH_2Cl_2 = 2:1, R_f = 0.29) did not give satisfactory results. Purification by *rec*-GPC gave **12b** as an orange solid (yield: 36%). ^{1}H NMR (400 MHz, $CDCl_3$) δ ppm 8.42 (m, 4H), 8.08 (s, 2H), 8.04 (s, 2H), 7.56 (d, *J* = 5.3 Hz, 2H), 2.89 (t, *J* = 7.9 Hz, 4H), 1.84–1.76 (m, 4H), 1.55–1.48 (m, 4H), 1.40–1.38 (m, 8H), 0.94 (t, *J* = 6.8 Hz, 3H), 0.93 (t, *J* = 6.8 Hz, 3H). MS (EI): *m/z* (%) = 624.2 (100) [$M^{+\cdot}$], 540.1 (27), 483.0 (24), 460.2 (7). ICR MALDI: *m/z* (M^+) (calcd: 624.1761 g/mol) found: 624.1756. Chemical formula: $C_{36}H_{36}N_2S_4$. M_w: 624.18 g/mol.

2,5-Bisdithienyl-9,10-dihexyldithieno[3,2-*a*:2',3'-*c*]phenazine (13): The synthesis was carried out analogous to the synthesis of **6** (petroleum ether 40/60:CH_2Cl_2 = 2:1, R_f = 0.62). **13** was obtained as a red solid (yield: 26%). ^{1}H NMR (400 MHz, $CDCl_3$) δ ppm 8.36 (s, 2H), 8.15 (s, 2H), 7.25 (dd, 2H, *J* = 5.0 Hz), 7.16 (dd, 2H, *J* = 3.4 Hz), 7.08 (d, 2H, *J* = 3.6 Hz), 7.03 (dd, 2H, *J* = 5.0 Hz), 6.98 (d, 2H, *J* = 3.7 Hz), 2.88 (t, *J* = 8.0 Hz, 4H), 1.83–1.77 (m, 4H), 1.53–1.51 (m, 4H), 1.45–1.35 (m, 8H), 0.94 (t, *J* = 6.0 Hz 6H). ^{13}C NMR (100 MHz, $CDCl_3$) δ ppm 137.8, 136.8, 136.8, 136.7, 136.7, 134.9, 128.0, 125.8, 125.3, 124.8, 124.8, 124.3, 124.3, 124.1, 124.0, 120.1, 33.0, 31.8, 29.8, 29.6, 22.7, 14.1. MS (EI): *m/z* (%) = 788.1 (7) [$M^{+\cdot}$], 624.1 (100). ICR MALDI *m/z* (M^+) (calcd: 788.1516 g/mol) found: 788.1515. Chemical formula: $C_{44}H_{40}N_2S_6$. M_w: 789.19 g/mol.

2,5-(3-Hexyl)bisthienyl-9,10-dihexyldithieno[3,2-*a*:2',3'-*c*]phenazine (14): 10b (100 mg, 0.16 mmol), 3-hexyl-thiophene-2-boronic acid pinacol ester (190 mg, 0.65 mmol), aqueous Cs_2CO_3 solution (1M; 2 mL) and toluene (5.5 mL) were purged with argon for 1.5–2 h. $Pd(PPh_3)_4$ (10 mg, 8.0 µmol) was added and the reaction mixture was heated to reflux for 20 h, cooled to rt, poured into water and extracted with dichloromethane. The organic layer was washed successfully with water (2×), saturated aqueous $NaHCO_3$ solution and brine, then dried over $MgSO_4$, filtered and evaporated. Purification of the crude product on silica gel (petroleum ether 40/60:CH_2Cl_2 = 2:1, R_f = 0.64) did not give satisfactory results. Purification by *rec*-GPC gave 25 mg (0.03 mmol, 19%) of **14** as an orange solid. ^{1}H NMR (400 MHz, $CDCl_3$) δ ppm 8.47 (s, 2H), 8.13 (s, 2H), 7.29 (d, *J* = 5.2 Hz, 2H), 7.02 (d, *J* = 5.2 Hz,

2H), 2.95 (t, *J* = 6.4 Hz, 4H), 2.91 (t, *J* = 6.4 Hz, 4H), 1.85–1.78 (m, 4H), 1.78–1.72 (m, 4H), 1.53–1.50 (m, 4H), 1.47–1.44 (m, 4H), 1.40–1.38 (m, 8H), 1.37–1.34 (m, 8H), 0.93 (t, *J* = 5.6 Hz, 6H), 0.89 (t, *J* = 5.6 Hz, 6H). ^{13}C NMR (125 MHz, $CDCl_3$) *δ* ppm 145.2, 140.9, 140.6, 139.0, 135.5, 135.0, 134.6, 130.2, 130.2, 127.2, 124.8, 123.0, 33.1, 31.8, 31.7, 30.6, 30.5, 29.7, 29.5, 29.4, 29.2, 22.6, 22.6, 14.11. MS (EI): *m/z* (%) = 792.4 (100) ($M^{+\cdot}$), 721.4 (9). ICR MALDI: *m/z* (M^+) (calcd: 792.3639 g/mol) found: 792.3634. Chemical formula: $C_{48}H_{60}N_2S_4$. M_w: 793.26 g/mol.

2,5-(5-Hexyl)-bisdithienyl-9,10-dihexyldithieno[3,2-*a*:2’,3’-*c*]phenazine (15): 10b (200 mg, 0.3 mmol), 5-hexyl-dithiophene-2-boronic acid pinacol ester (487 mg, 1.3 mmol), Aliquat 336 (60 mg, 0.15 mmol), aqueous Na_2CO_3 solution (2M; 4.5 mL) and toluene (20 mL) were purged with argon for 1.5–2 h. $Pd(PPh_3)_4$ (10 mg, 9 µmol) was added and the reaction mixture was heated to reflux for 20h, cooled to rt, poured into water and extracted with dichloromethane. The organic layer was successfully washed with aqueous 0.1 M HCl, saturated aqueous Na_2CO_3 solution and brine, then dried over $MgSO_4$, filtered and evaporated. Purification of the crude product on silica gel (petroleum ether 40/60:CH_2Cl_2 = 2:1, R_f = 0.56) gave 65 mg (0.06 mmol, 21%) of **15** as a red solid. ^{1}H NMR (400 MHz, $CDCl_3$) *δ* ppm 8.00 (s, 2H), 7.86 (s, 2H), 6.94 (dd, 4H, *J* = 2.8 Hz, *J* = 3.2 Hz), 6.84 (d, 2H, *J* = 3.6 Hz), 6.68 (d, 2H, *J* = 3.6 Hz), 2.81 (t, 4H, *J* = 7.6 Hz), 2.65 (t, 4H, *J* = 7.6 Hz), 1.76–1.68 (m, 8H), 1.45–1.36 (m, 24H), 0.95 (t, *J* = 6.8 Hz, 6H), 0.94 (t, *J* = 6.8 Hz, 6H). MS (MALDI-pos, *DCTB*): *m/z* (%) = 956.3 (100) ($M^{+\cdot}$). ICR MALDI: *m/z* (M^+) (calcd: 956.3393 g/mol) found: 956.3388. Chemical formula: $C_{56}H_{64}N_2S_6$. M_w: 957.51 g/mol.

2,5-Dithienyl-9,10-dimethyldithieno[2,3-*a*:3’,2’-*c*]phenazine (16): The synthesis was carried out analogous to the synthesis of **6** (petroleum ether 40/60:$CHCl_3$ = 1:4, R_f = 0.40). **16** was obtained as an orange solid (yield: 49%). ^{1}H NMR (400 MHz, $CDCl_3$) *δ* ppm 8.08 (s, 2H), 7.77 (s, 2H), 7.46 (dd, 2H, *J* = 0.9Hz, *J* = 3.6 Hz), 7.37 (dd, 2H, *J* = 0.9 Hz, *J* = 5.1 Hz), 7.13 (dd, 2H, *J* = 3.6 Hz, *J* = 5.1 Hz), 2.57 (s, 6H). MS (EI): *m/z* (%) = 484.0 (100) [$M^{+\cdot}$], 469.0 (5), 402.0 (5), 242.0 (20). EI HRMS: *m/z* (M^+) (calcd: 484.0191 g/mol) found: 484.0185. Chemical formula: $C_{26}H_{16}N_2S_4$. M_w: 484.68 g/mol.

2,5-Bisbithienyl-9,10-dimethyldithieno[2,3-*a*:3',2'-*c*]phenazine (17a): The synthesis was carried out analogous to the synthesis of **6**. **17a** was obtained as a red solid (recrystallization from *o*-xylene) (yield: 15%). ^{1}H NMR (400 MHz, DMSO-d$_6$) *δ* ppm 8.29 (s, 2H), 7.99 (s, 2H), 7.61 (dd, *J* = 0.8Hz, *J* = 5.0 Hz, 2H), 7.55 (d, *J* = 3.8 Hz, 2H), 7.43 (dd, *J* = 1.0 Hz, *J* = 3.5 Hz, 2H), 7.38 (d, *J* = 3.8 Hz, 2H), 7.17 (dd, *J* = 3.7 Hz, *J* = 4.9 Hz, 2H), 2.52 (s, 6H). MS: (MALDI-pos, *DCTB*) *m/z* (%) = 648.0 (100) [$M^{+\cdot}$]. ICR MALDI. *m/z* (M^+) (calcd: 647.9945 g/mol) found: 647.9946. Chemical formula: $C_{34}H_{20}N_2S_6$. M_w: 648.93 g/mol.

2,5-Bisbithienyl-9,10-dihexyldithieno[2,3-*a*:3',2'-*c*]phenazine (17b): The synthesis was carried out analogous to the synthesis of **6.** The crude product of **17b** was obtained as a red solid (yield: 19%). An analytical sample could only be obtained by purification via *rec*-GPC. ^{1}H NMR (400 MHz, CDCl$_3$) *δ* ppm 8.03 (s, 2H), 7.72 (s, 2H), 7.32 (d, *J* = 3.76 Hz, 2H), 7.27 (dd, *J* = 5.11, 1.08 Hz, 2H), 7.24 (dd, *J* = 3.57, 1.00 Hz, 2H), 7.16 (d, *J* = 3.77 Hz, 2H), 7.06 (dd, *J* = 3.77, 5.08 Hz, 2H), 2.86 (t, *J* = 7.78 Hz, 4H), 1.86–1.72 (m, 4H), 1.54–1.45 (m, 4H), 1.42–1.35 (m, 8H), 0.93 (t, *J* = 6.48 Hz, 6H). MS (MALDI-pos, DCTB): *m/z* (%) = 788.2 (100) [$M^{+\cdot}$]. ICR MALDI: *m/z* (M^+) (calcd: 788.1510 g/mol) found: 788.1511. Chemical formula: $C_{44}H_{40}N_2S_6$. M_w: 789.19 g/mol.

2,5-(5-Hexyl)bisdithienyl-9,10-dihexyldithieno[2,3-*a*:3',2'-*c*]phenazine (18): 11b (455 mg, 0.74 mmol), 5-hexyl-dithiophene-2-boronic acid pinacol ester (835 mg, 2.22 mmol), Aliquat 336 (100 mg, 0.25 mmol), aqueous Na_2CO_3 solution (2M; 5 mL) and toluene (15 mL) were purged with argon for 1.5–2 h. Pd(PPh$_3$)$_4$ (80 mg, 70 µmol) was added and the reaction mixture was heated to reflux for 20 h, cooled to rt, poured into water and extracted with dichloromethane. The organic layer was washed successfully with water (2×), saturated aqueous $NaHCO_3$ solution and brine, dried over $MgSO_4$, filtered and evaporated. Purification of the crude product on silica gel (petroleum ether 40/60:CH_2Cl_2 = 2:1, R_f = 0.42) gave 400 mg (0.42 mmol, 56%) of **18** as a red solid. ^{1}H NMR (400 MHz, CD_2Cl_2) *δ* ppm 7.57 (s, 2H), 6.92 (d, *J* = 3.45 Hz, 2H), 6.91 (s, 2H), 6.86 (d, *J* = 3.63 Hz, 2H), 6.76 (d, *J* = 3.67 Hz, 2H), 6.70 (d, *J* = 3.47 Hz, 2H), 2.83 (t, *J* = 7.65 Hz, 4H), 2.53 (t, *J* = 7.47 Hz, 4H), 1.77–1.62 (m, 16H), 1.42–1.35 (m, 16H), 0.96 (t, *J* = 6.56 Hz , 6H), 0.95 (t, *J* = 7.11 Hz , 6H). ^{13}C NMR (100 MHz, CD_2Cl_2) *δ* ppm 145.7, 144.2, 140.3, 139.9, 137.6, 137.6, 134.9, 134.8,

134.4, 132.9, 125.6, 125.2, 124.9, 123.4, 123.1, 117.2, 32.6, 32.1, 31.7, 31.7, 30.3, 29.8, 29.3, 29.0, 22.9, 22.8, 14.1, 14.0. MS (MALDI-pos, *DCTB*): *m/z* (%) = 956.3 (100) [$M^{+\cdot}$]. ICR MALDI: *m/z* (M^+) (calcd: 956.3388 g/mol) found: 956.3388. Chemical formula: $C_{56}H_{64}N_2S_6$. M_w: 957.51 g/mol.

Quaterthiophene (19): The synthesis was carried out analogous to the synthesis of **6** (petroleum ether 40/60:CH_2Cl_2 = 5:1, R_f = 0.48). **19** was obtained as a yellow solid (yield: 48%). ^{1}H NMR (400 MHz, $CDCl_3$) δ ppm 7.22 (dd, ^3J = 5.2 Hz, ^4J = 1.2 Hz, 2H), 7.18 (dd, ^3J = 5.2 Hz, ^4J = 1.2 Hz, 2H), 7.08 (dd, ^3J = 5.2 Hz, ^4J = 3.8 Hz, 4H), 7.02 (dd, ^3J = 5.2 Hz, ^4J = 3.6 Hz, 2H). ^{13}C NMR (100 MHz, $CDCl_3$) δ ppm 137.0, 136.3, 135.8, 127.9, 124.5, 124.3, 124.2, 123.7. MS (EI): *m/z* (%) = 330.0 (20) [$M^{+\cdot}$], 272.2 (50), 165.0 (20), 149.0 (100). EI HRMS: *m/z* (M^+) (calcd: 329.9660 g/mol) found: 329.9661. Chemical formula: $C_{16}H_{10}S_4$. M_w: 330.51 g/mol.

Sexithiophene (20): The synthesis was carried out analogous to the synthesis of **6** (petroleum ether 40/60:CH_2Cl_2 = 5:1, R_f = 0.45). **20** was obtained as an orange solid (yield: 15%). NMR measurements were not possible due to its low solubility. MS (EI) *m/z* (%) = 493.8 (100) [$M^{+\cdot}$], 329.9 (10), 246.9 (20). EI HRMS: *m/z* (M^+) (calcd: 493.9414 g/mol) found: 493.9422. Chemical formula: $C_{24}H_{14}S_6$. M_w: 494.76 g/mol.

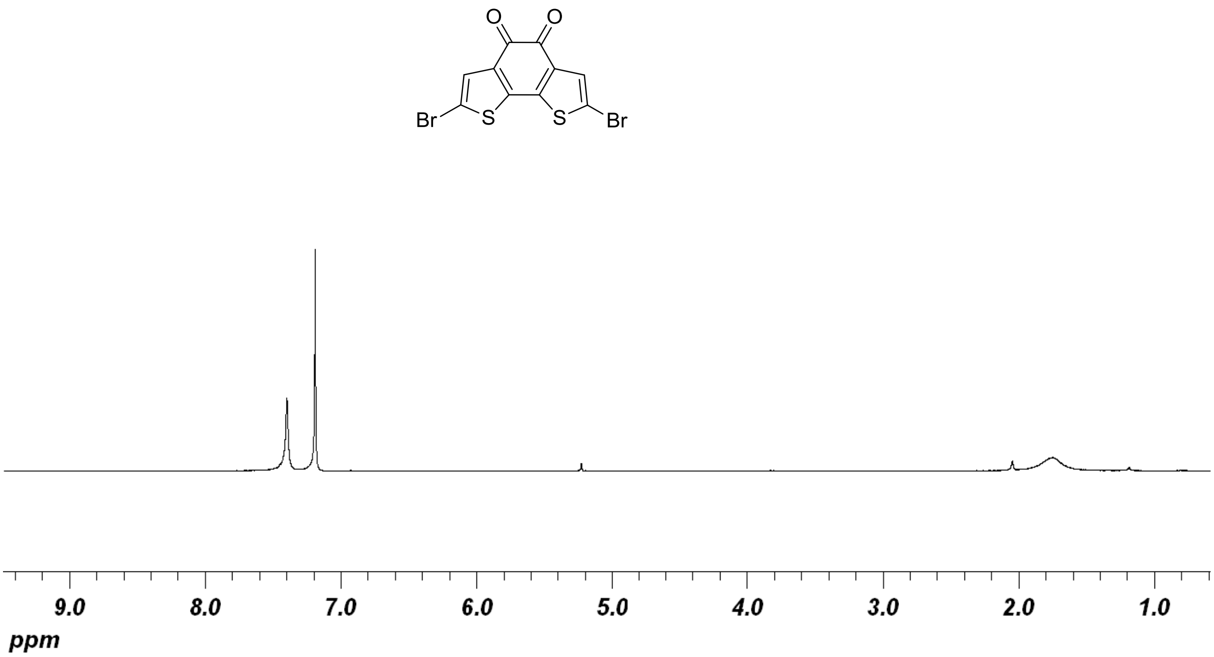

Figure 1: ^{1}H NMR spectrum of **4** (400 MHz, $CDCl_3$).

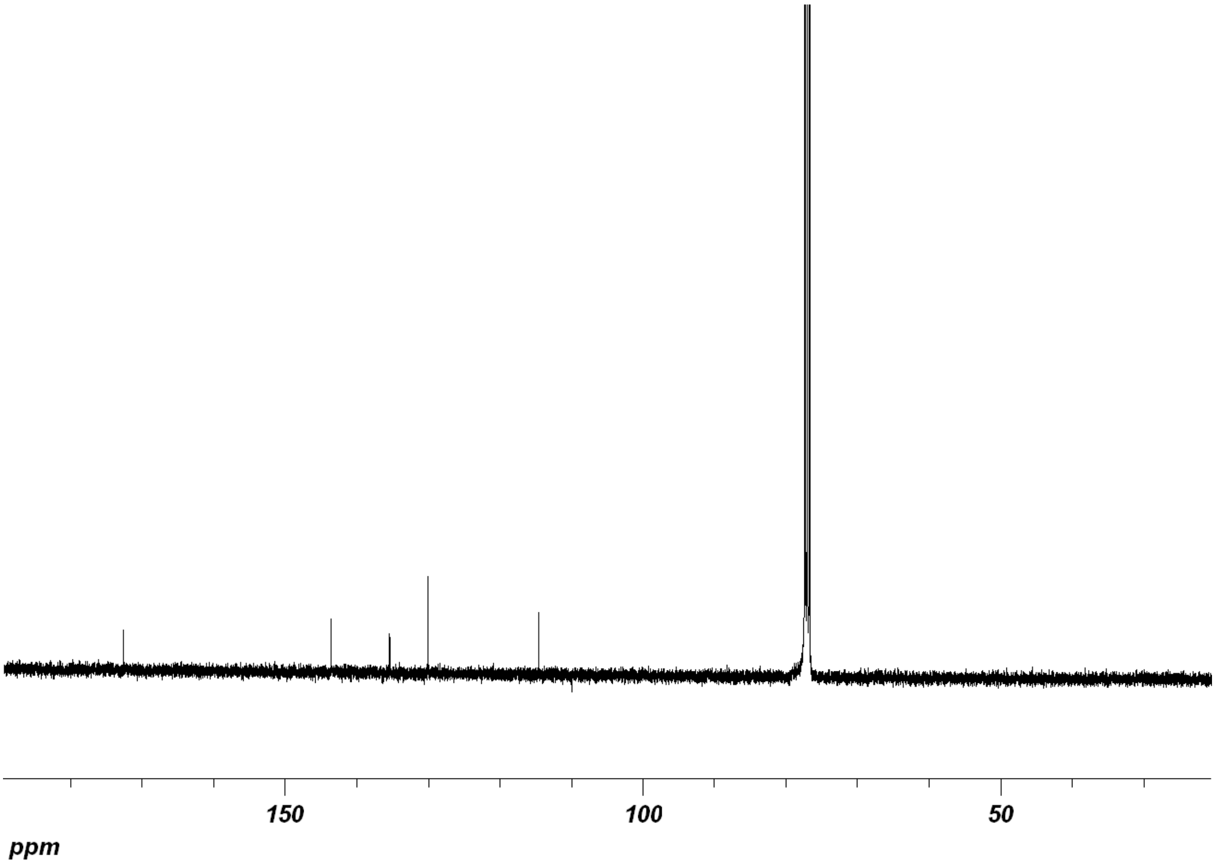

Figure 2: ^{13}C NMR spectrum of **4** (100 MHz, $CDCl_3$).

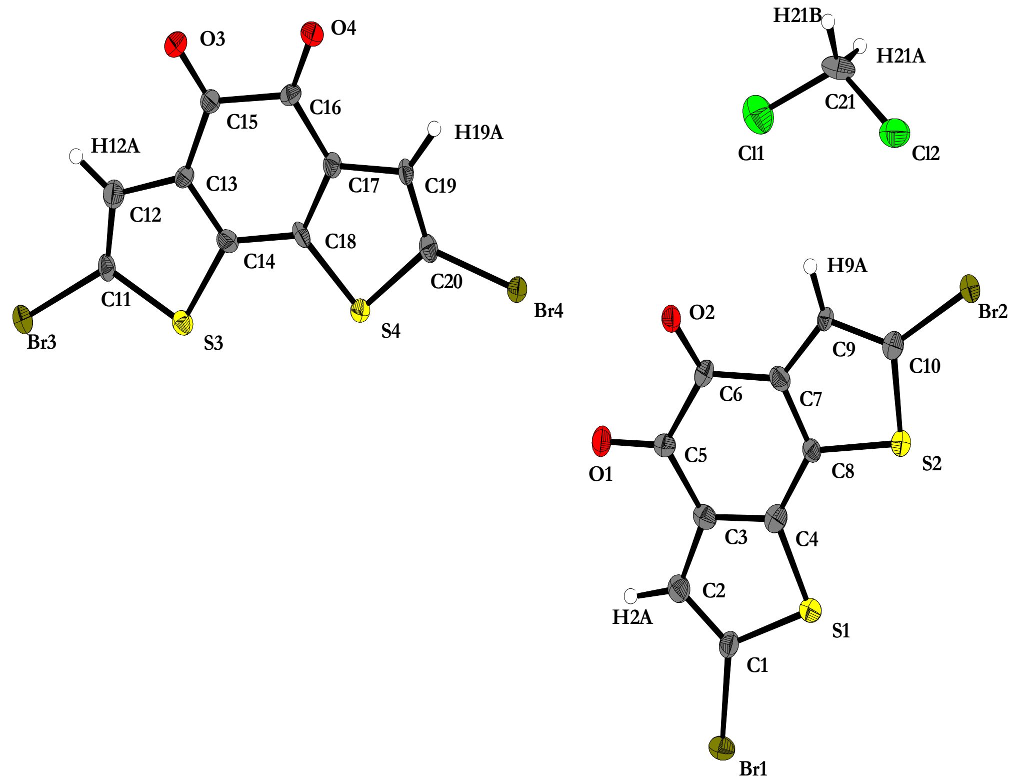

Asymmetric unit of **4**.

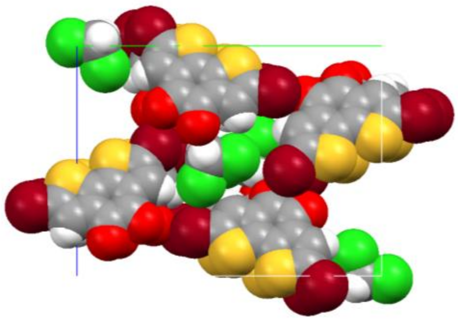

Packing of **4**; view along a.

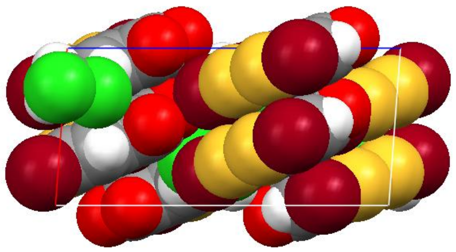

Packing of **4**; view along b.

Figure 3: Crystal structures of **4**.

Crystal data of **4**:

Device Type	STOE IPDS 2T
Empirical formula	$C_{21}H_6Br_4Cl_2O_4S_4$
Formula weight	841.04
Temperature	123(2) K
Wavelength	0.71073 Å
Crystal system, space group	Monoclinic, P 2_1/c
Unit cell dimensions	a = 7.5928(8) Å α = 90°
	b = 20.9792(11) Å β = 94.236(8)°
	c = 15.9792(13) Å γ = 90°
Volume	2538.4(4) $Å^3$
Z, Calculated density	4, 2.201 gcm^{-3}
Absorption coefficient	6.911 mm^{-1}
F(000)	1608
Crystal size	0.3 x 0.02 x 0.02 mm
Theta range for data collection	2.32 to 28.00°
Limiting indices	-10<=h<=9, -27<=k<=27, -18<=l<=21
Reflections collected / unique	19770 / 5987 [R_{int} = 0.1351]
Completeness to theta = 28.00	98.0%
Absorption correction	Semi-empirical from equivalents
Max. and min. transmission	0.68355 and 0.528
Refinement method	Full-matrix least-squares on F^2
Data / restraints / parameters	5987 / 0 / 316
Goodness-of-fit on F^2	0.895
Final R indices [I>2sigma(I)]	R_1 = 0.0672, wR_2 = 0.1211
R indices (all data)	R_1 = 0.1545, wR_2 = 0.1478
Largest diff. peak and hole	0.889 and -1.138 eA^{-3}

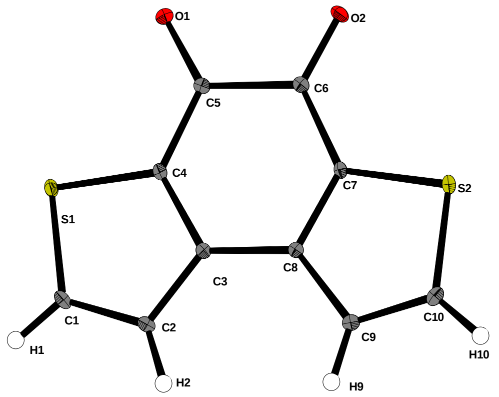

Asymmetric unit of **7**.

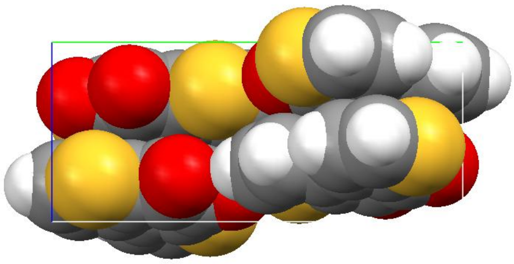

Packing of **7**; view along a.

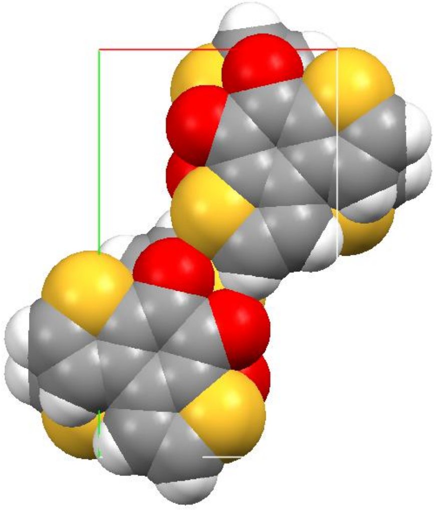

Packing of **7**; view along c.

Figure 4: Crystal structures of **7**.

Crystal data of **7**:

Device Type	Nonius KappaCCD
Empirical formula	$C_{10}H_4O_2S_2$
Formula weight	220.27
Temperature	123(2) K
Wavelength	0.71073 Å
Crystal system, space group	Monoclinic, P 2_1/c
Unit cell dimensions	a = 8.9233(4) Å α = 90°
	b = 14.8008(8) Å β = 102.935(3)°
	c = 6.6258(2) Å γ = 90°
Volume	852.88(7) $Å^3$
Z, Calculated density	4, 1.715 gcm^{-3}
Absorption coefficient	0.585 mm^{-1}
F(000)	448
Crystal size	0.6 x 0.429 x 0.161 mm
Theta range for data collection	2.75 to 27.99°
Limiting indices	-11<=h<=10, -17<=k<=19, -8<=l<=7
Reflections collected / unique	7685 / 2029 [R_{int} = 0.0424]
Completeness to theta = 27.99	98.4%
Absorption correction	Semi-empirical from equivalents
Max. and min. transmission	0.91287 and 0.79308
Refinement method	Full-matrix least-squares on F^2
Data / restraints / parameters	2029 / 0 / 128
Goodness-of-fit on F^2	1.044
Final R indices [I>2sigma(I)]	R_1 = 0.0272, wR_2 = 0.0699
R indices (all data)	R_1 = 0.0376, wR_2 = 0.0736
Extinction coefficient	0.0068(13)
Largest diff. peak and hole	0.292 and -0.316 $eÅ^{-3}$

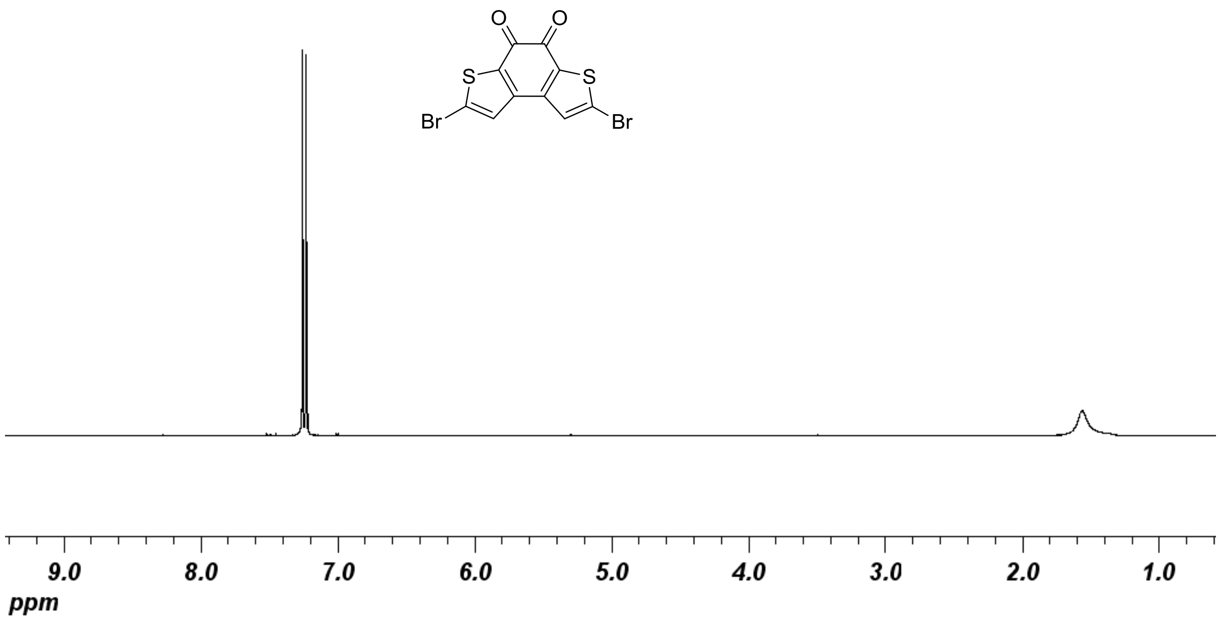

Figure 5: ^{1}H NMR spectrum of **8** (400 MHz, $CDCl_3$).

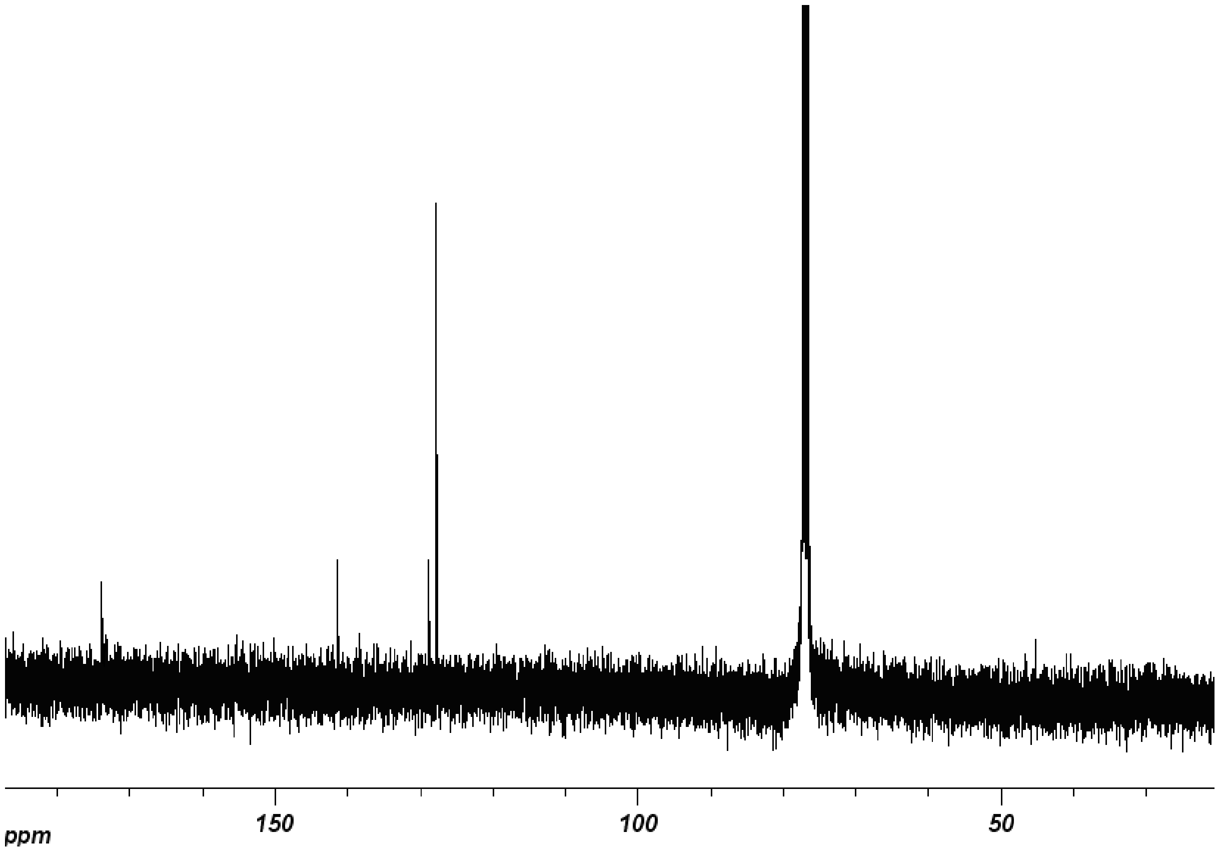

Figure 6: ^{13}C NMR spectrum of **8** (100 MHz, $CDCl_3$).

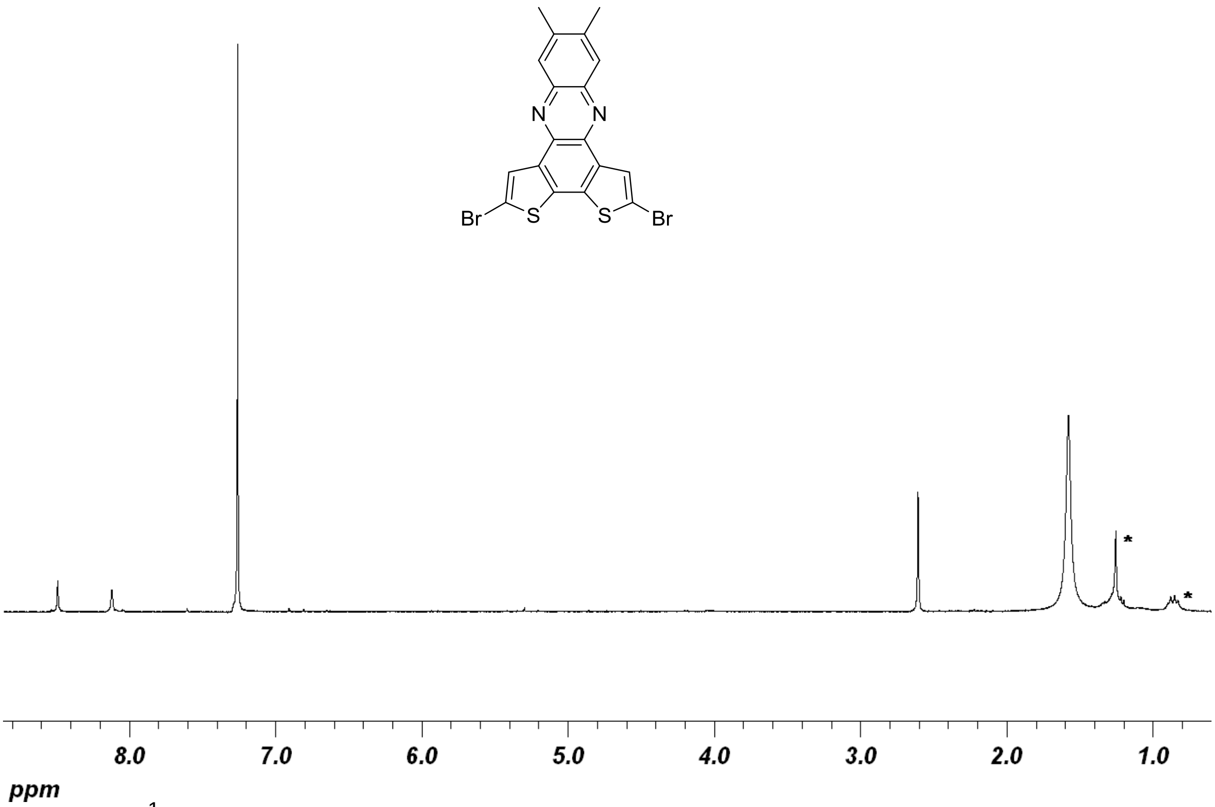

Figure 7: ^{1}H NMR spectrum of **10a** (400 MHz, $CDCl_3$).

*The spectrum includes some residual petroleum ether.

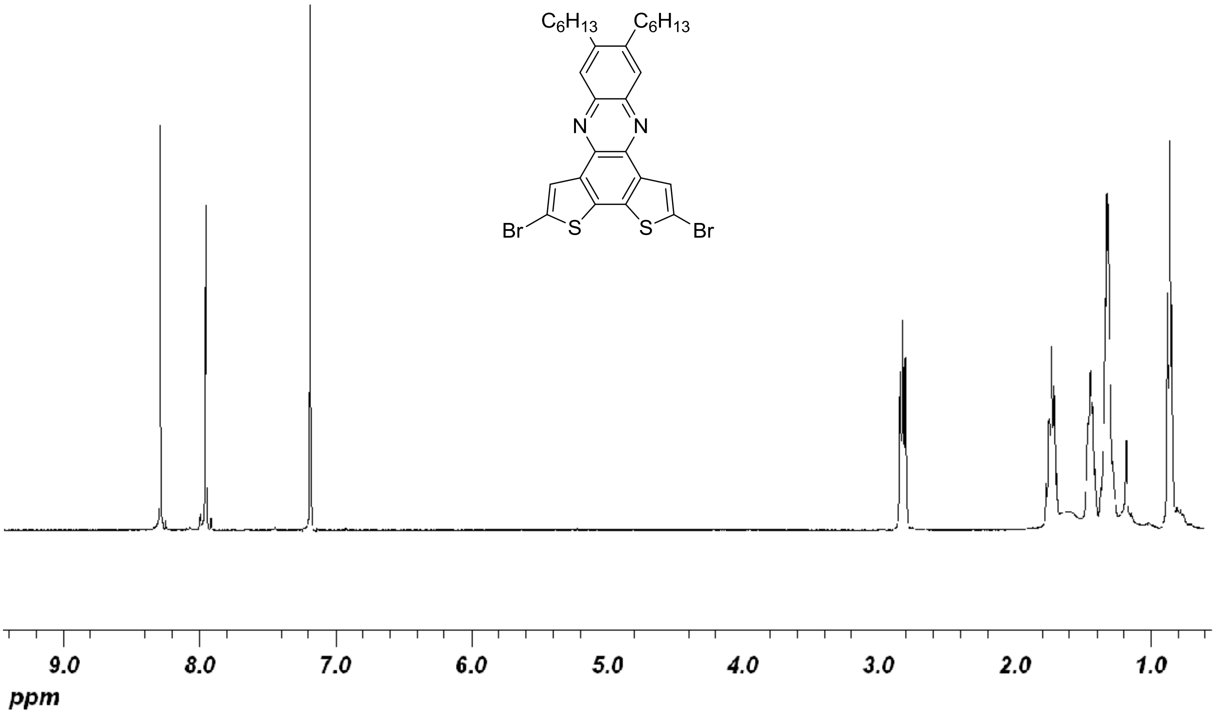

Figure 8: ^{1}H NMR spectrum of **10b** (400 MHz, $CDCl_3$).

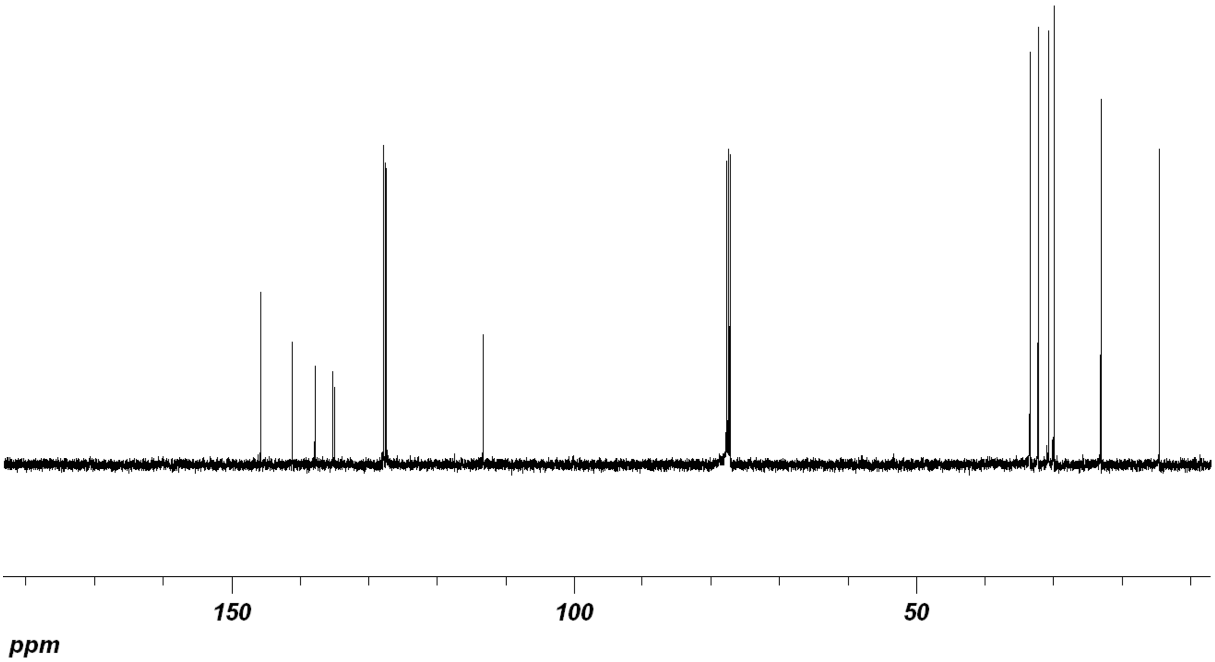

Figure 9: ^{13}C NMR spectrum of **10b** (75 MHz, $CDCl_3$).

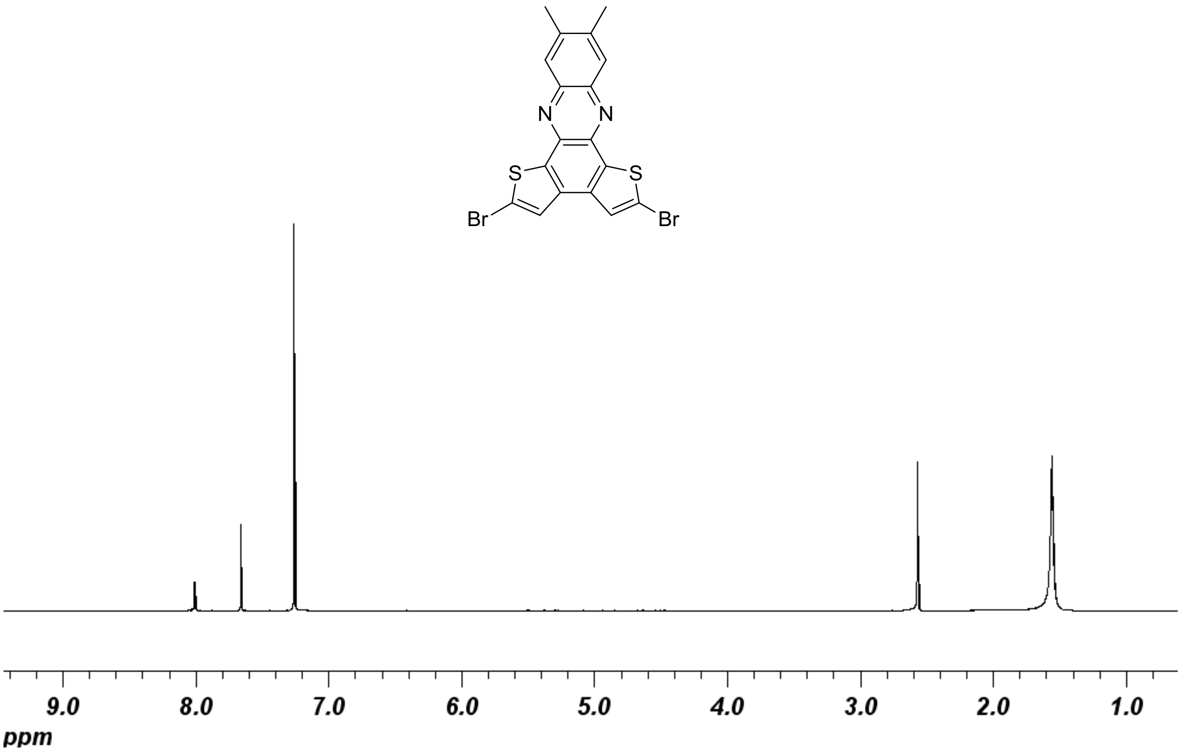

Figure 10: ^{1}H NMR spectrum of **11a** (400 MHz, $CDCl_3$).

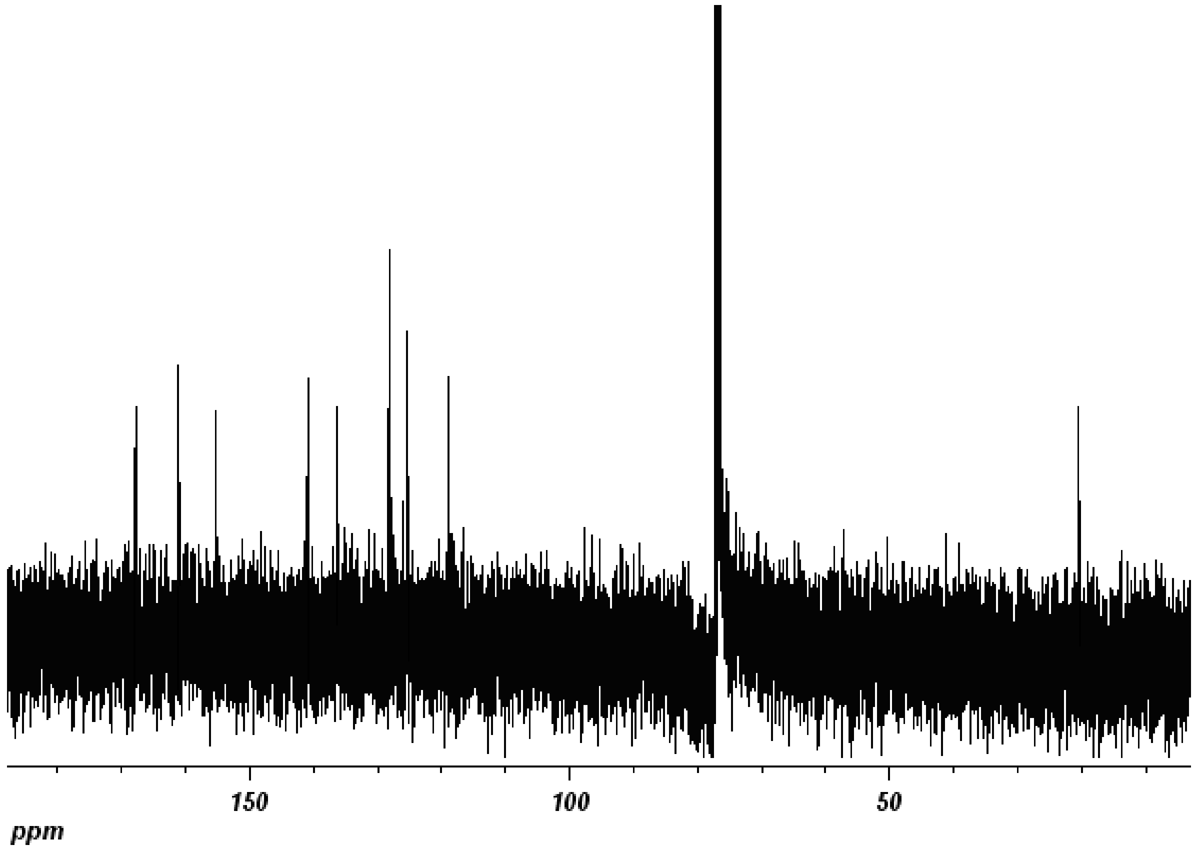

Figure 11: ^{13}C NMR spectrum of **11a** (100 MHz, $CDCl_3$).

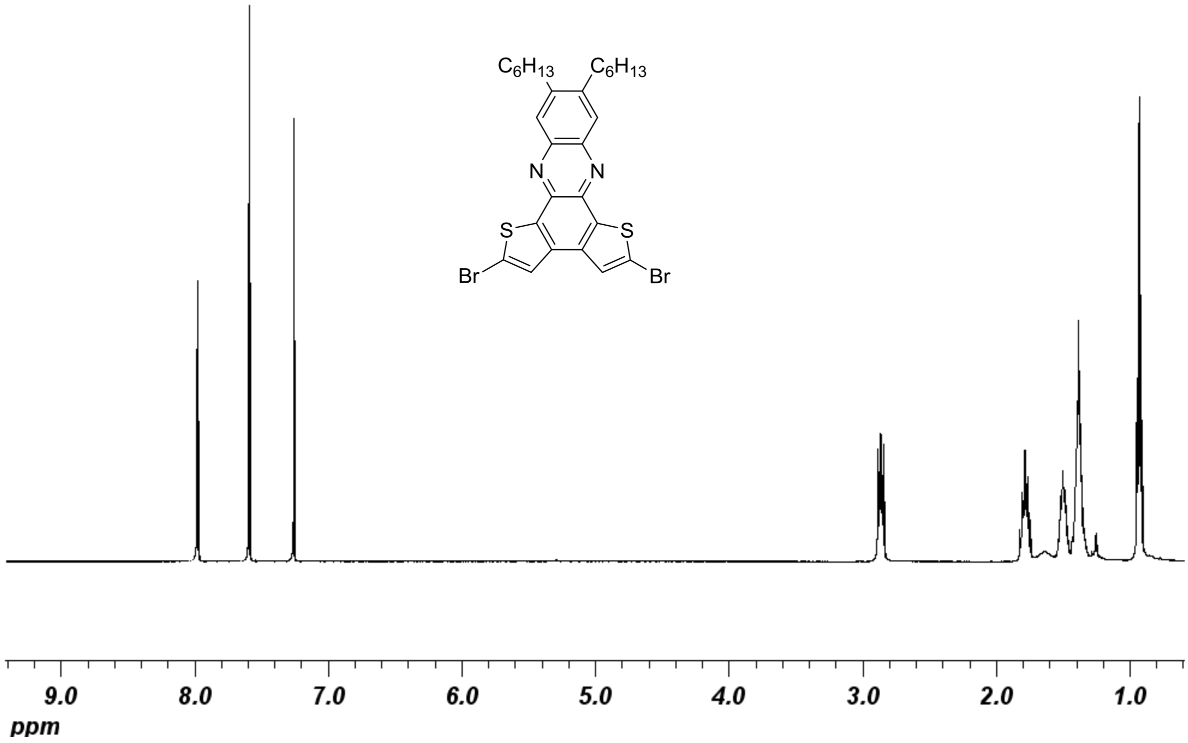

Figure 12: ^{1}H NMR spectrum of **11b** (400 MHz, $CDCl_3$).

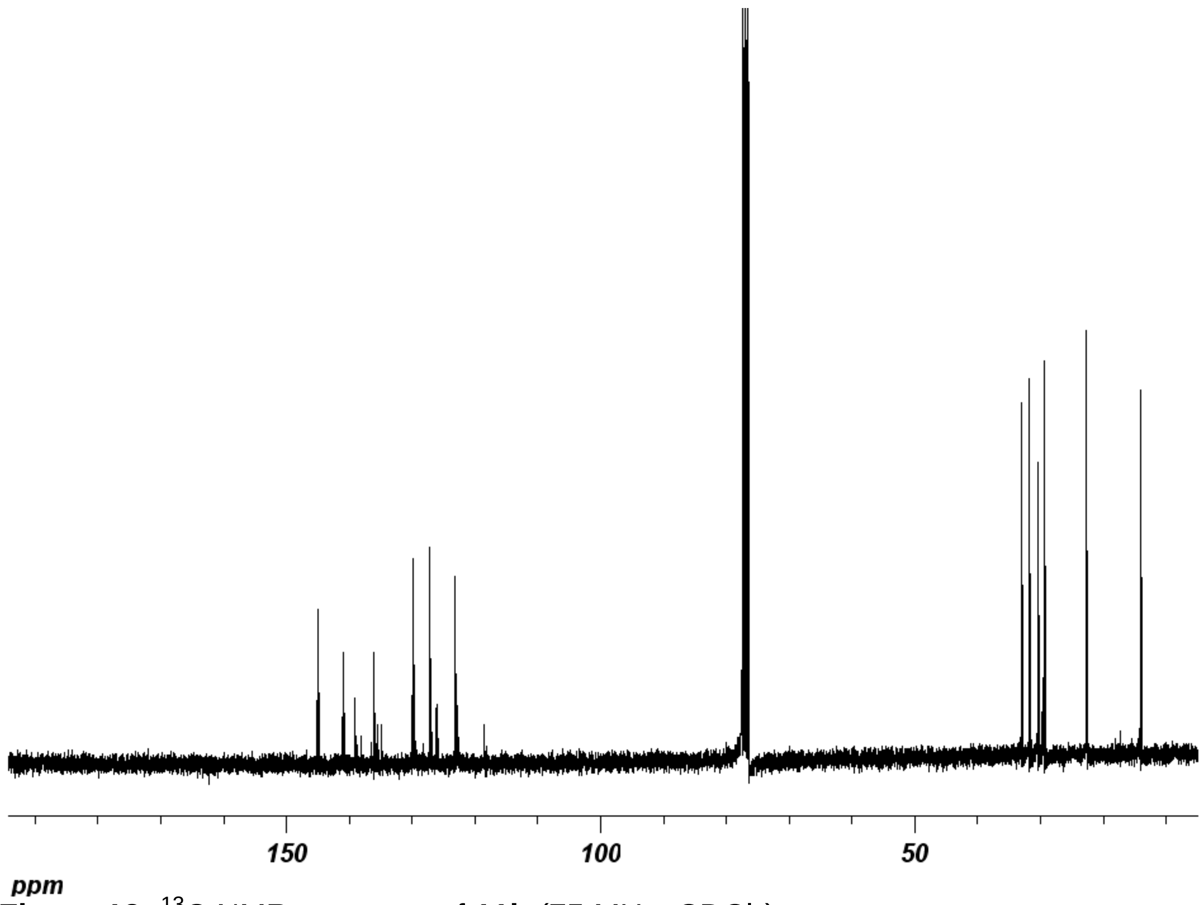

Figure 13: ^{13}C NMR spectrum of **11b** (75 MHz, $CDCl_3$).

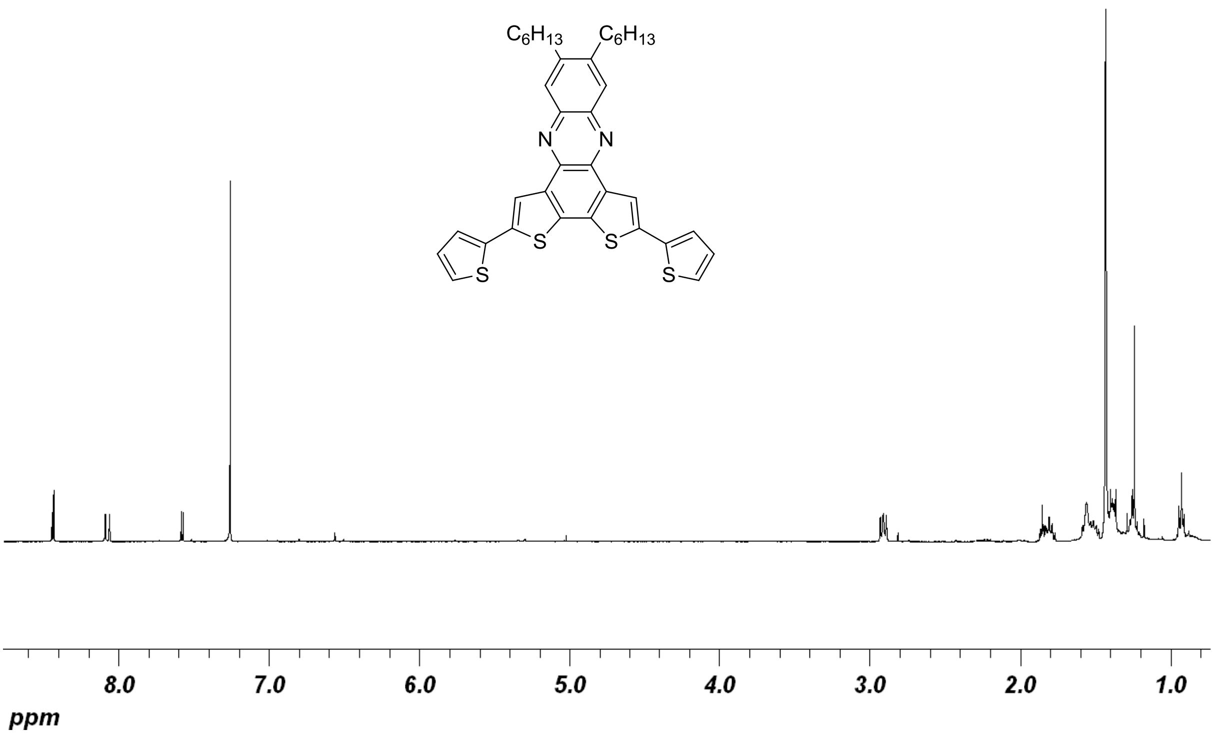

Figure 14: ^{1}H NMR spectrum of **12b** (400 MHz, $CDCl_3$).

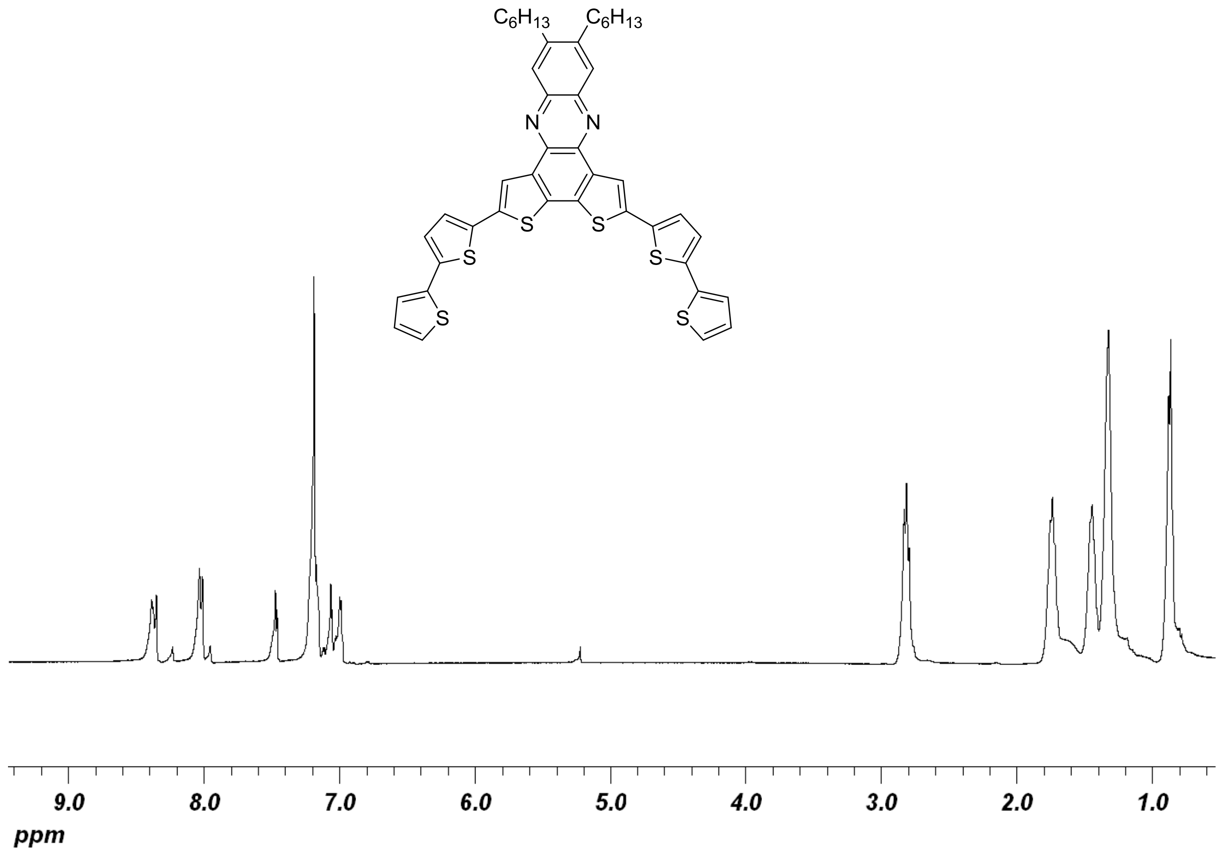

Figure 15: ^{1}H NMR spectrum of **13** (400 MHz, $CDCl_3$).

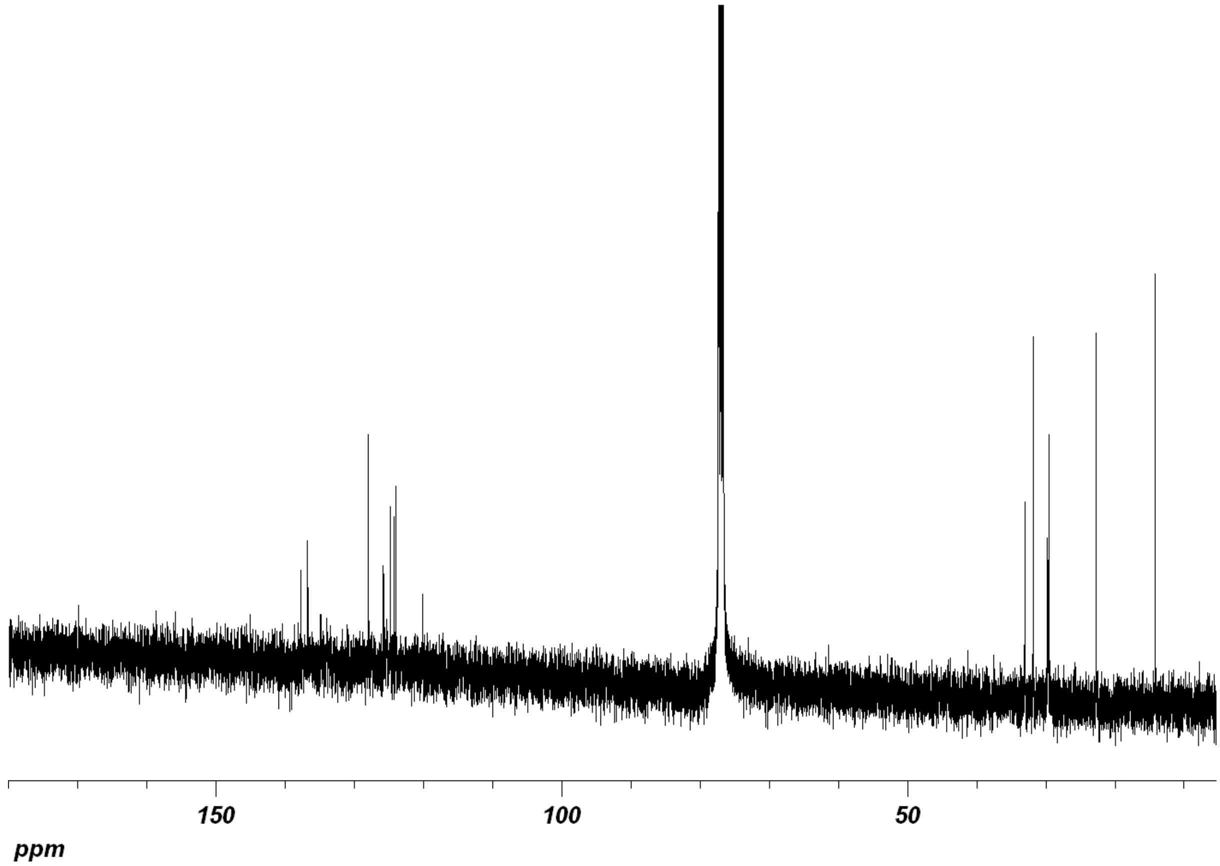

Figure 16: ^{13}C NMR spe..ctrum of **13** (100 MHz, $CDCl_3$).

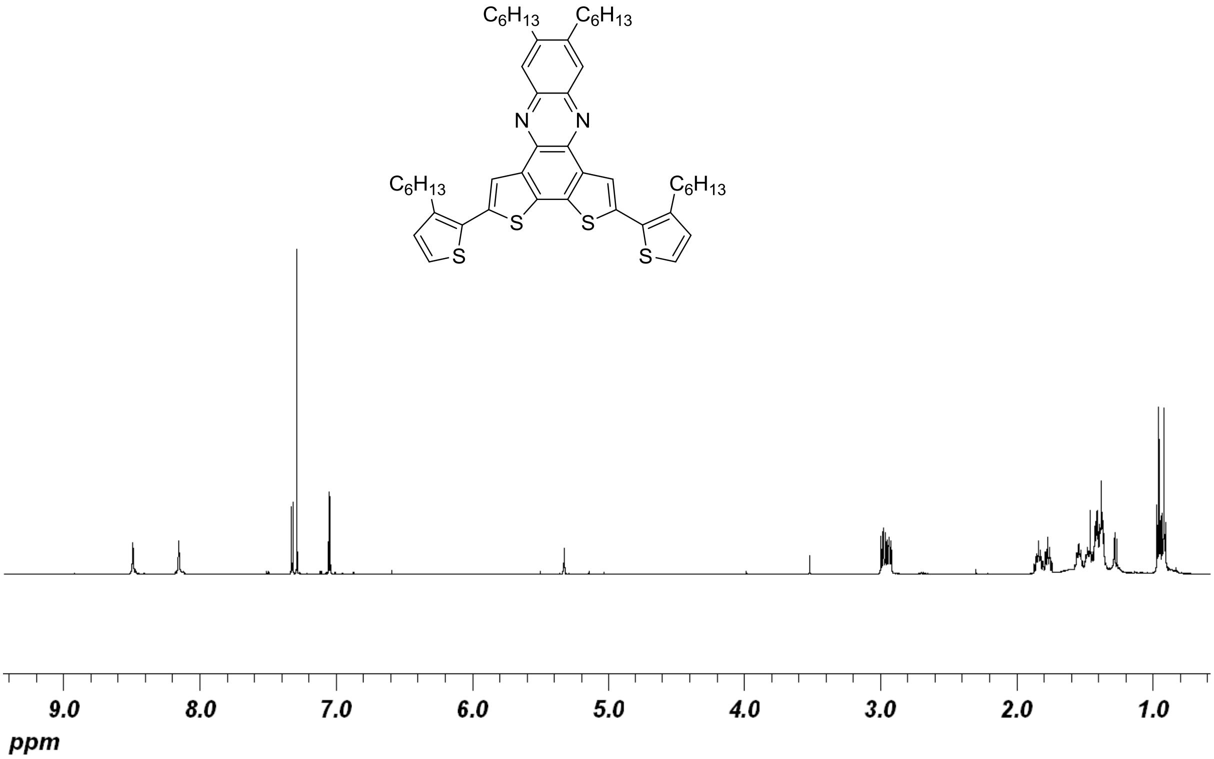

Figure 17: ^{1}H NMR spectrum of **14** (400 MHz, $CDCl_3$).

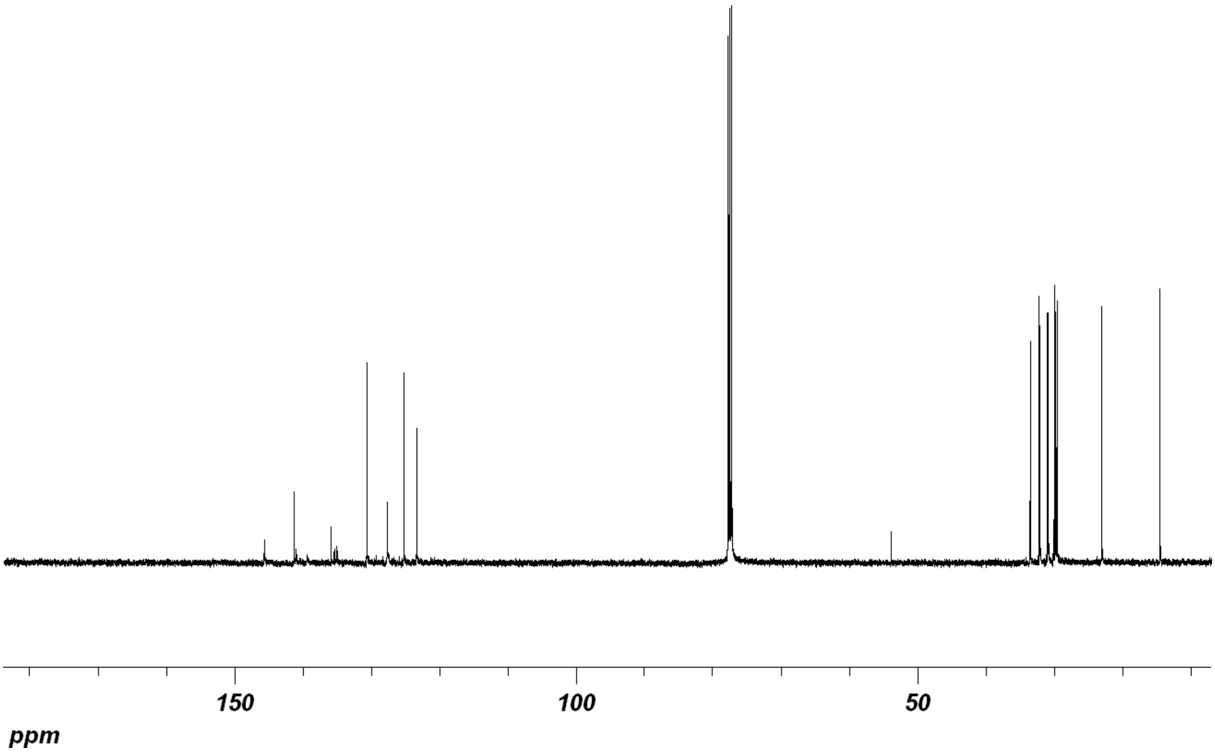

Figure 18: ^{13}C NMR spectrum of **14** (125 MHz, $CDCl_3$).

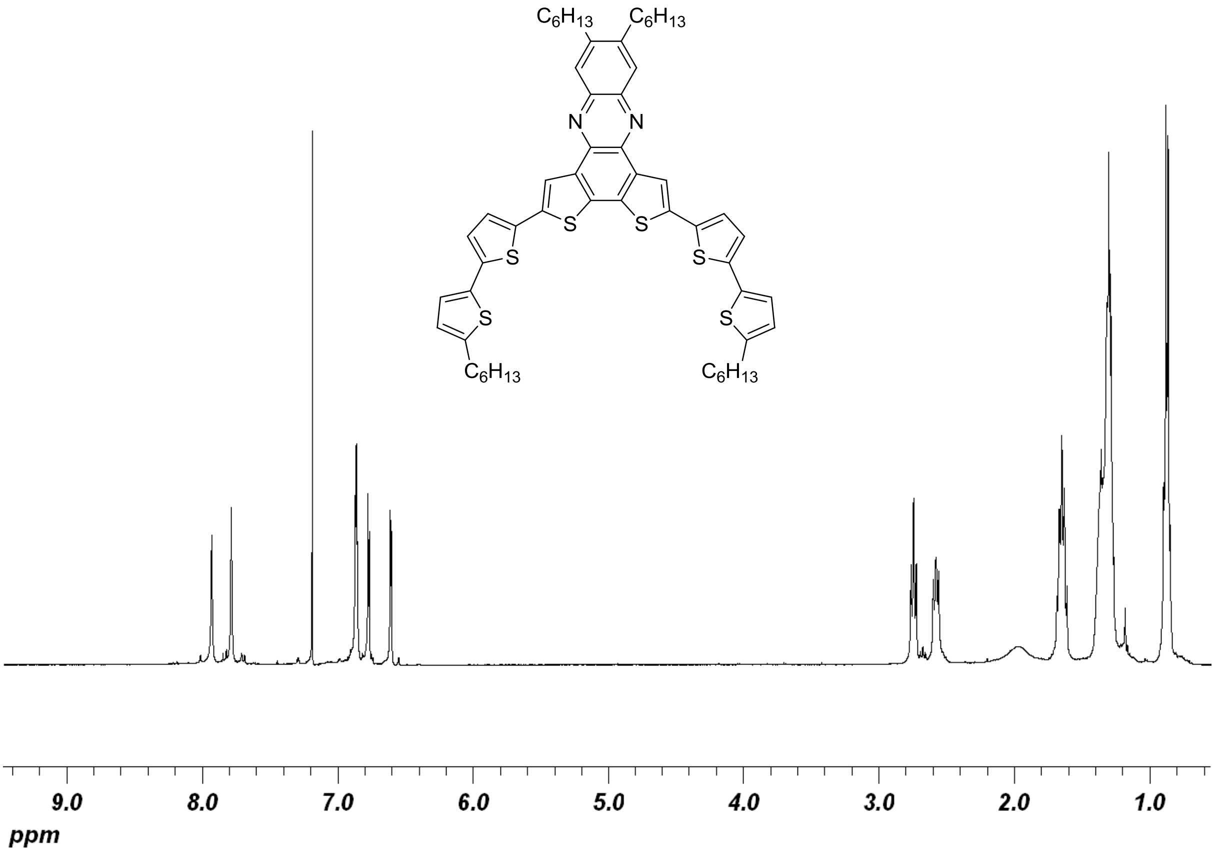

Figure 19: ^{1}H NMR spectrum of **15** (400 MHz, $CDCl_3$).

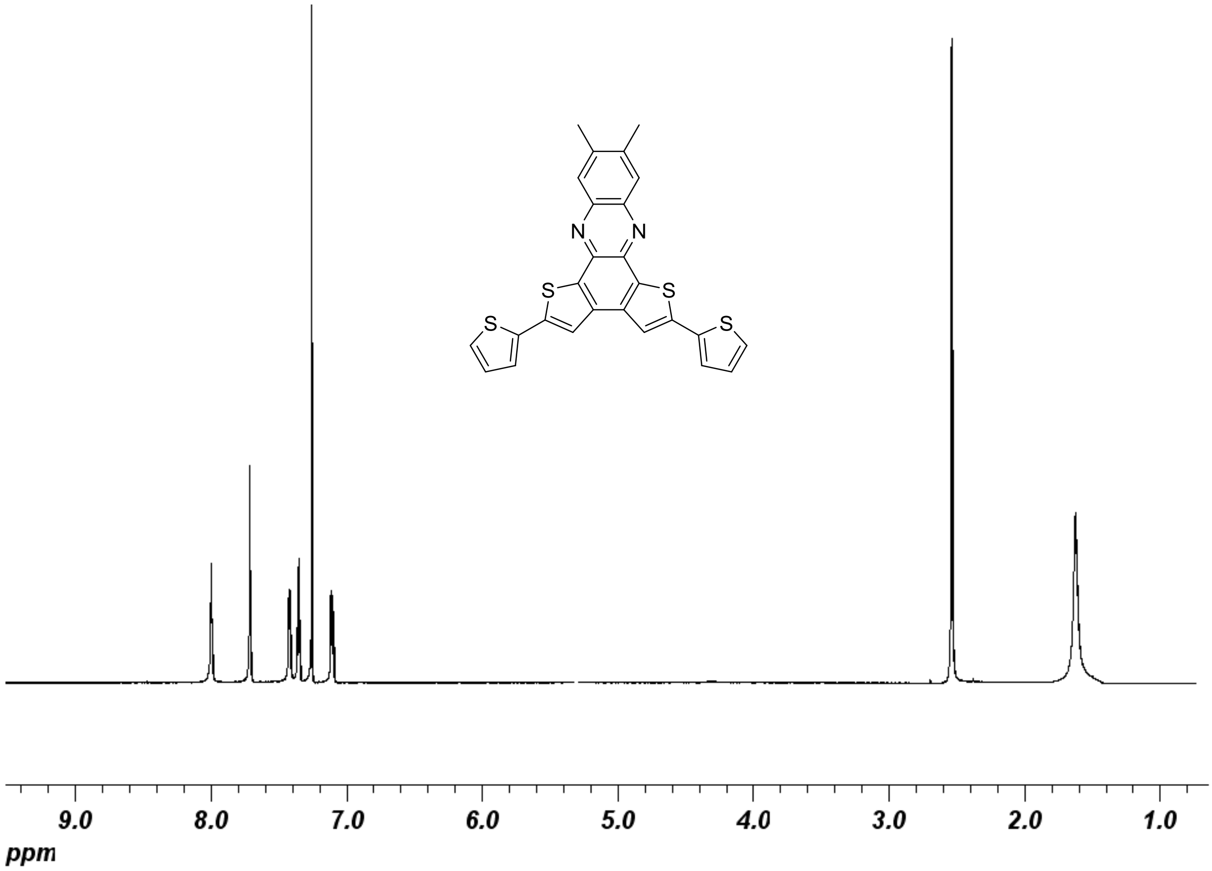

Figure 20: ^{1}H NMR spectrum of **16** (400 MHz, $CDCl_3$).

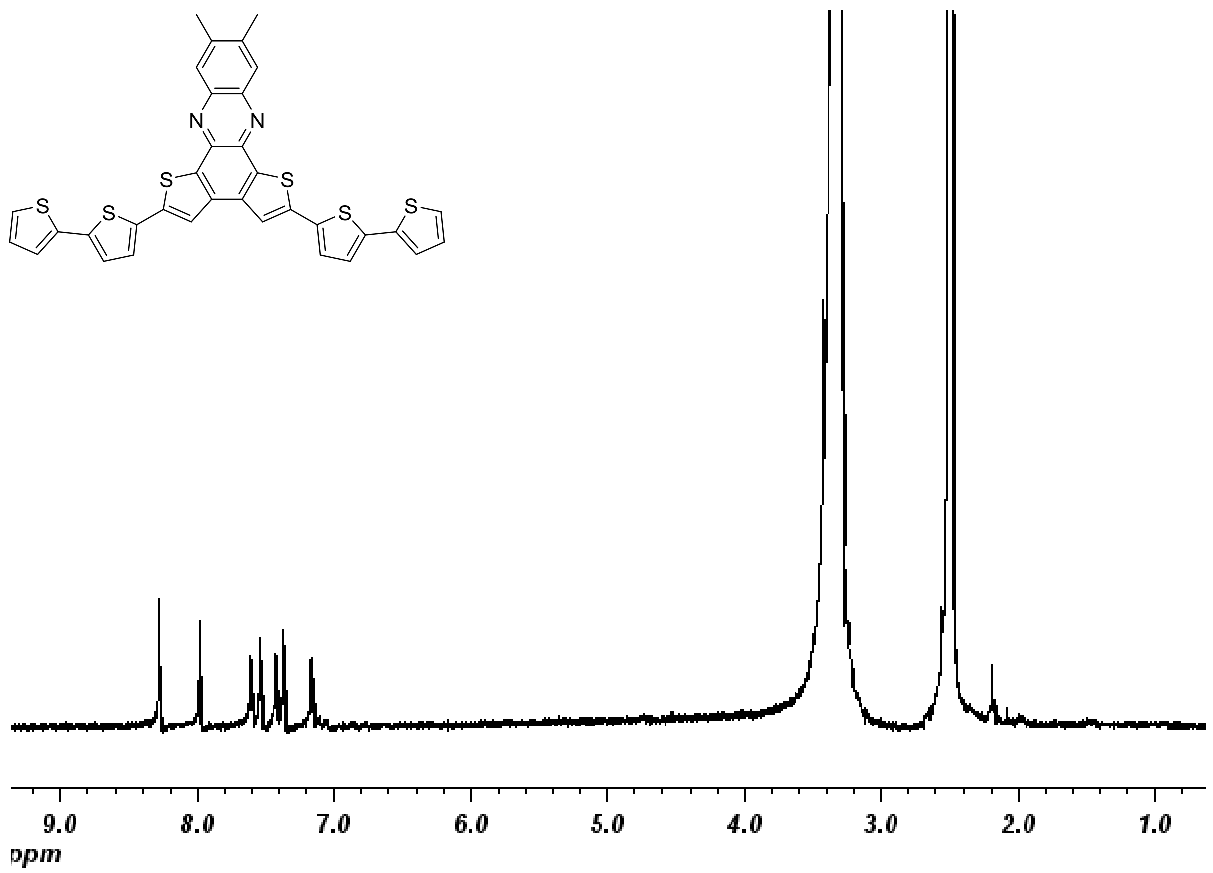

Figure 21: ^{1}H NMR spectrum of **17a** (400 MHz, DMSO-d$_6$).

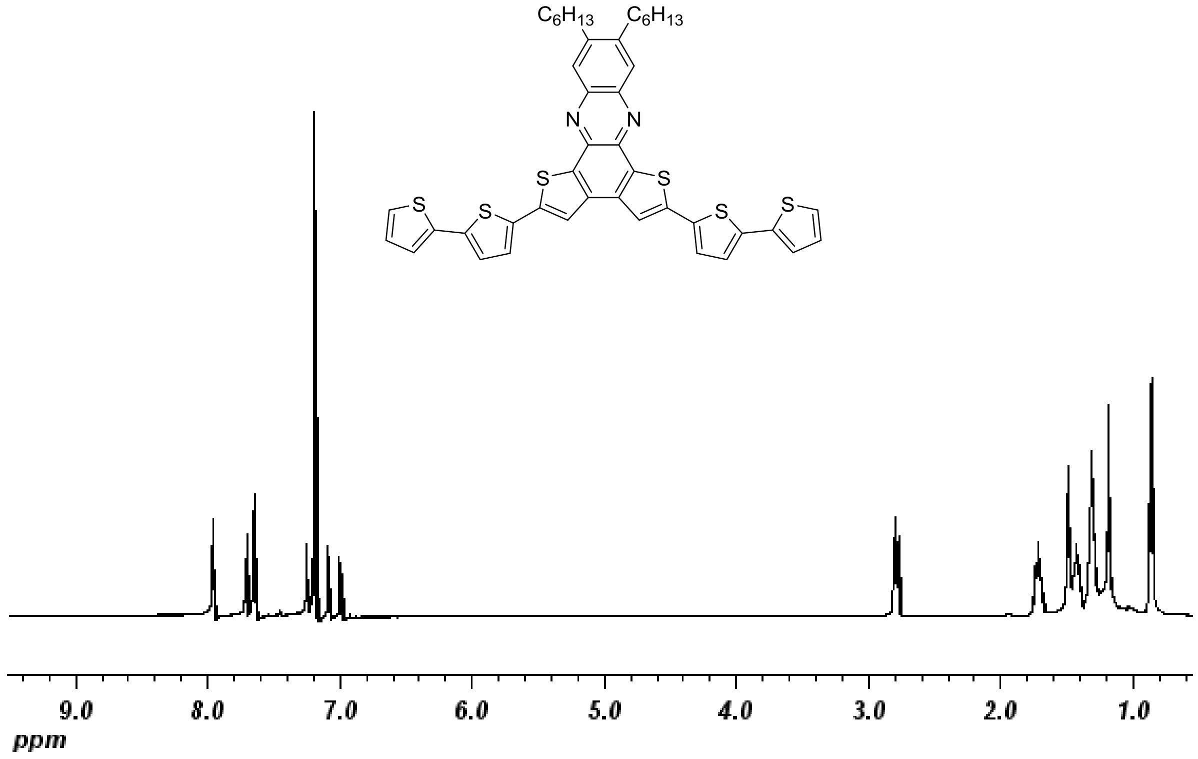

Figure 22: ^{1}H NMR spectrum of **17b** (400 MHz, $CDCl_3$).

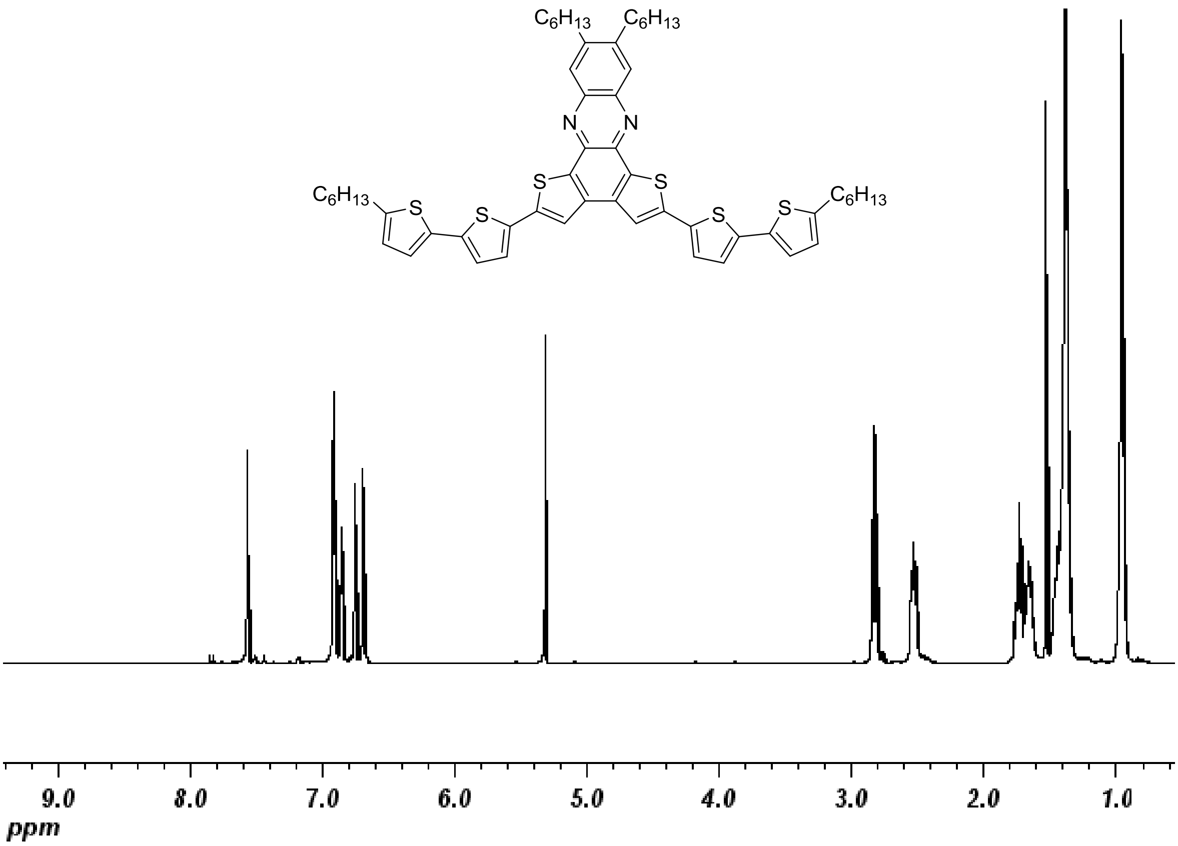

Figure 23: ^{1}H NMR spectrum of **18** (400 MHz, CD_2Cl_2).

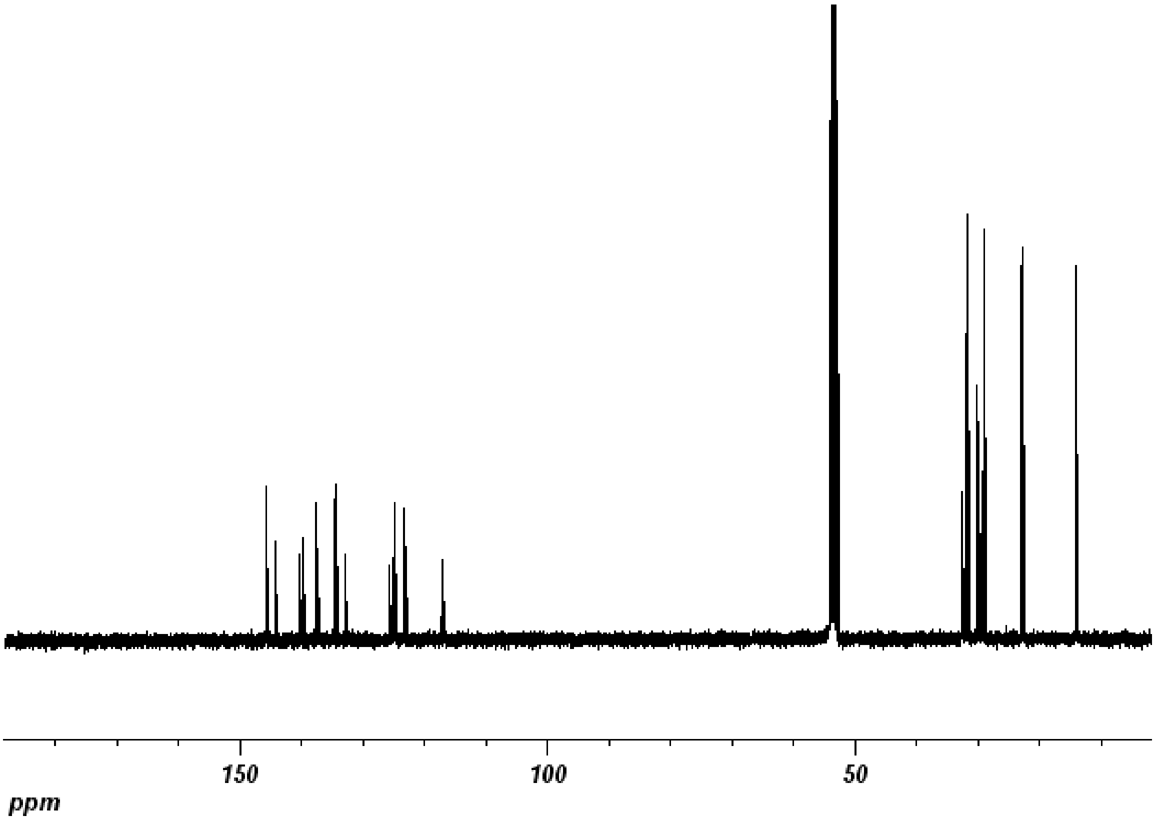

Figure 24: ^{13}C NMR spectrum of **18** (100 MHz, CD_2Cl_2).

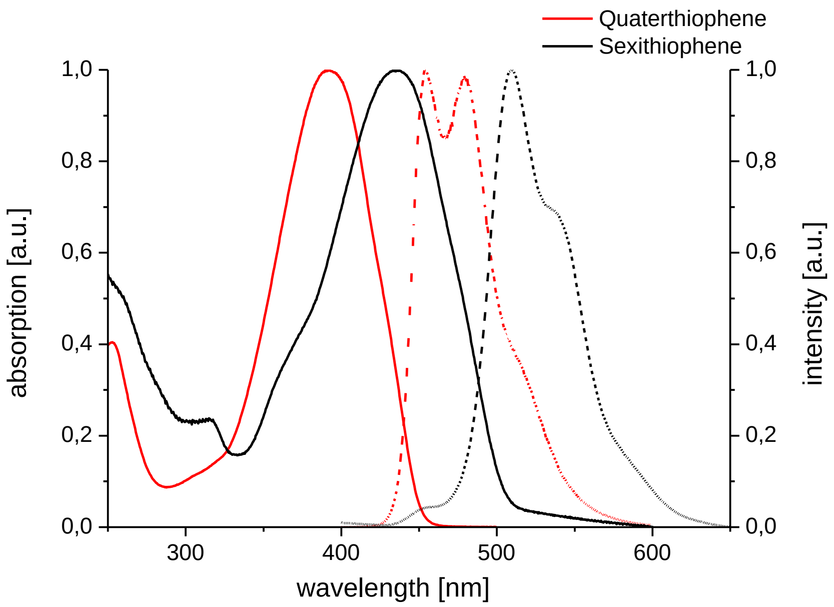

Figure 25: Absorption (solid line) and emission (dotted line) spectra of quaterthiophene (red) and sexithiophene (black) in CH_2Cl_2.

References

1. Nyberg, K. *Acta Chem. Scand.* **1969,** *23,* 1087–1089. doi:10.3891/acta.chem.scand.23-1087
2. Phillips, K. E. S.; Katz, T. J.; Jockusch, S.; Lovinger, A. J.; Turro, N. J. *J. Am. Chem. Soc.* **2001,** *123,* 11899–11907. doi:10.1021/ja011706b
3. Zhang, F.; Bai, S.; Yap, G. P.; Fox, J. M. *J. Am. Chem. Soc.* **2005,** *127,* 10590–10599. doi:10.1021/ja050886c
4. Blessing, R. H. *Acta Crystallogr., Sect. A* **1995,** *51,* 33–38. doi:10.1107/S0108767394005726
5. Sheldrick, G. M. *SHELXS97* and *SHELXL97*, University of Göttingen: Germany, 1997.
6. Brandenburg, K. *DIAMOND 2.1c*, Crystal Impact GbR: Bonn, Germany, 1999.
